# A network meta-analysis of secondary attack rates of COVID-19 in different contact environments

**DOI:** 10.1017/S0950268821002223

**Published:** 2021-10-05

**Authors:** Xunying Zhao, Ziqiong Shen, Litao Sun, Long Cheng, Mengyuan Wang, Xiaofan Zhang, Bin Xu, Lulu Tian, Yunqi Miao, Xueyao Wu, Kun Zou, Jiayuan Li

**Affiliations:** 1 Department of Epidemiology and Health Statistics, West China School of Public Health and West China Fourth Hospital, Sichuan University, Chengdu 610041, China; 2 Centre for Medical Education, Cardiff University, Cardiff, UK; 3 Department of Child, Adolescent and Maternal Health, West China School of Public Health and West China Fourth Hospital, Sichuan University, Chengdu, China; 4 West China Research Centre for Rural Health Development, Sichuan University, Chengdu, China; 5 HEOA Group, Institute for Healthy Cities, Sichuan University, Chengdu, China

**Keywords:** Contact environment, COVID-19, network meta-analysis, secondary attack rate

## Abstract

As the corona virus disease 2019 (COVID-19) pandemic continues around the world, understanding the transmission characteristics of COVID-19 is vital for prevention and control. We conducted the first study aiming to estimate and compare the relative risk of secondary attack rates (SARs) of COVID-19 in different contact environments. Until 26 July 2021, epidemiological studies and cluster epidemic reports of COVID-19 were retrieved from SCI, Embase, PubMed, CNKI, Wanfang and CBM in English and Chinese, respectively. Relative risks (RRs) were estimated in pairwise comparisons of SARs between different contact environments using the frequentist NMA framework, and the ranking of risks in these environments was calculated using the surface under the cumulative ranking curve (SUCRA). Subgroup analysis was performed by regions. Thirty-two studies with 68 260 participants were identified. Compared with meal or gathering, transportation (RR 10.55, 95% confidence interval (CI) 1.43–77.85), medical care (RR 11.68, 95% CI 1.58–86.61) and work or study places (RR 10.15, 95% CI 1.40–73.38) had lower risk ratios for SARs. Overall, the SUCRA rankings from the highest to the lowest were household (95.3%), meal or gathering (81.4%), public places (58.9%), daily conversation (50.1%), transportation (30.8%), medical care (18.2%) and work or study places (15.3%). Household SARs were significantly higher than other environments in the subgroup of mainland China and sensitive analysis without small sample studies (<100). In light of the risks, stratified personal protection and public health measures need to be in place accordingly, so as close contacts categorising and management.

## Introduction

The world is still in the pandemic of corona virus disease 2019 (COVID-19) since its outbreak in December 2019 [1]. Although vaccination is available and implemented in many countries, public health measures are still essential for disease control and prevention. The transmission of COVID-19 is primarily through air and saliva during close contact [2]. Long-term contact with infected persons and short-term contact with symptomatic patients are associated with high risks of transmission [3].

Understanding the secondary attack rates (SARs) among diverse contact environments is meaningful for making corresponding close contacts' management plans. Epidemiological studies have been conducted on the SARs in different contact environments during the COVID-19 epidemic. However, the reported SARs varied largely across original studies. For example, the reported household SAR ranged from 1.87% to 55.56% [4, 5]. Several systematic reviews and traditional meta-analyses have also been conducted. Some reviews reported the estimate of pooled SARs of COVID-19 in households, which varied from 16.6% to 21.1% [6–8]. Besides, Tian and Huo [9] and Thompson *et al*. [10] also estimated SARs in other contact environments, such as social gathering settings, schools, workplaces, healthcare facilities and so on, with pooled SARs varying from 1% to 6%. These systematic reviews estimated the pooled SARs in different contact environments by subgroup analysis. However, pairwise comparisons and relative risks (RRs) of SARs among multiple contact environments have not been systematically estimated yet. Therefore, the understanding and evidence are lacking on whether there is a statistical difference between the SARs of two or more contact environments.

Network meta-analysis (NMA) can be an effective method to solve this problem. Compared with traditional meta-analysis, it can directly and indirectly compare multiple treatments and interventions, and more accurately evaluate the relative effects of different comparison groups to draw conclusions [11]. NMA can not only compare the effects of treatments and interventions in pairs, but also get the relative ranking of these effects [12]. For example, Hutton *et al*. [13] conducted an NMA on the risk of lymphoid tissue disorders and other cancers in kidney transplant patients by using the results of randomised controlled trials and observational studies. Most epidemiological studies of infectious diseases are observational studies. When a certain research problem in observational studies has multiple groups, the comparison of multi-arm groups can also be analysed using the principle of NMA.

Therefore, this study aimed to comprehensively compare the risk of SARs between different contact environments during the period of COVID-19 epidemic using the NMA.

## Materials and methods

### Data sources and searches

We searched English databases including PubMed, Embase and Web of Science, and Chinese databases including China National Knowledge Infrastructure (CNKI), Wanfang and China Biology Medicine disc (CBM) until 26 July 2021. Search terms included ‘novel corona*’ OR ‘new corona*’ OR ‘CoV’ OR ‘nCoV’ OR ‘COVID*’ OR ‘SARS-cov-2’ OR ‘SARS2’ combined with ‘Close contact*’ OR ‘family cluster’ OR ‘secondary attack rate’ OR ‘SAR’ OR ‘Transmission’ OR ‘contact trac*’. Subject words and free words (in title and abstract) were used in the search. The search strategy was first formulated in PubMed and then modified for other databases. The search strategies were presented in Supplementary Table S1. A hand search of the reference lists of included studies was made for more potentially eligible studies.

### Study selection

To be included, the primary studies must be retrospective analyses of epidemiological characteristics and clustered epidemic reports. The contact follow-up data of COVID-19 patients and their close contacts must exceed 7–14 days, including the total number of COVID-19 patients, the number of follow-up cases and contact environments (at least two contact environments). The SARs of the close contacts must be reported in the articles or could be calculated by manual statistics (the number of positive cases of the close contacts/the total number of the close contacts). The following types of studies were excluded, including duplicate reports (in which case the study with the largest sample size was included), meeting abstract, expert consensus and suggestions.

The inclusion or exclusion of each study was conducted by three reviewers (X.-Y. Zhao, Z.-Q. Shen or L.-T. Sun). First, abstracts and titles were read to exclude the obvious irrelevant studies. Then, full texts for potentially eligible studies were obtained and read against the inclusion and exclusion criteria. Finally, cross-checking was made among the three reviewers. Disagreements were determined by group discussions.

### Data extraction and quality assessment

A pre-designed data extraction table was used to extract data from studies including the first author, time of publication, study site, contact environments, the number of close contact management, the number of follow-up cases and the number of cases infected. The contact environments in each study were classified into seven well-defined categories.

The Joanna Briggs Institute (JBI) Critical Appraisal Tool for Case Series Study was used to evaluate the quality of studies [14]. Due to potential attrition bias introduced by the loss of follow-up, studies that did not conduct further tracking and polymerase chain reaction (PCR) testing of all identified close contacts were considered as low quality.

Data extraction and quality assessment were completed independently and cross-checked by two reviewers (X.-Y. Zhao, Z.-Q. Shen or L.-T. Sun). The classification of contact environments was defined according to the Guideline of COVID-19 Control and Prevention-Close Contact Management Procedure (7th ed.) and was adjusted by included studies [15, 16]. Finally, seven categories of contact environments were included in our study: household settings (Hou), public places (Pub), meal or gathering settings (Mea), transportation (Tra), daily conversation (Dai), work or study places (Wor) and medical care (Med) (Supplementary Table S3). The contact environments were classified by two researchers (X.-Y. Zhao and Z.-Q. Shen) independently. If contact environments reported in the study cannot be classified into one of the above seven types, the data were discarded. Any disagreement was resolved by discussion to reach a consensus.

### Definitions of measures

SAR is defined as the probability that close contacts of index cases become confirmed cases of a disease [17]. Relative risk (RR) is defined as the estimated relative risk between SARs of two different contact environments in pairs, calculated by the ratio of SARs between the two groups in the NMA [18] (the formula was as follows):
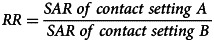


### Data synthesis and analysis

Pair-wise RRs of the SARs between two contact environments were estimated using frequentist NMA [12]. First, the consistency test was used to test the global inconsistency across the network [19, 20]. If *P* < 0.05, the inconsistency model was selected, otherwise the consistency model was applied to calculate the RRs. The side-splitting (node-splitting) method was further used to compare whether there was a significant difference between the direct comparison and the indirect comparison of each RR in two contact environments [21]. The surface under the cumulative ranking curve (SUCRA) was calculated and the probability of being the highest SAR among SARs of all contact environments was sorted in the descending order [11]. The *Q* test and *I*-squared (*I*^2^) metrics were used to evaluate the heterogeneity of the studies of each pairwise comparison. And publication bias was examined by funnel plots and Egger's test [12]. Subgroup analysis had been planned to assess whether there were differences in the RRs and ranking of the SARs among different regions. For sensitivity analysis, small sample studies (<100) and studies with low quality were excluded.

Traditional meta-analysis was also conducted as an additional analysis to estimate the pooled SARs for each contact environment. *Q* test was used to test the heterogeneity among studies, and *I*^2^ was applied to quantify the heterogeneity. If *I*^2^ ≤ 50% or *P* > 0.1, the fixed-effect model was used, otherwise the random-effect model was selected [22]. Publication bias was examined by funnel plots and Egger's test [23].

All analyses were performed using Stata 15.1 SE, employing network package [12] and metan package [24]. The significance level *α* was 0.05. The PROSPERO number of this study is CRD42020206576.

## Results

### Characteristics of included studies

**Fig. 1.**
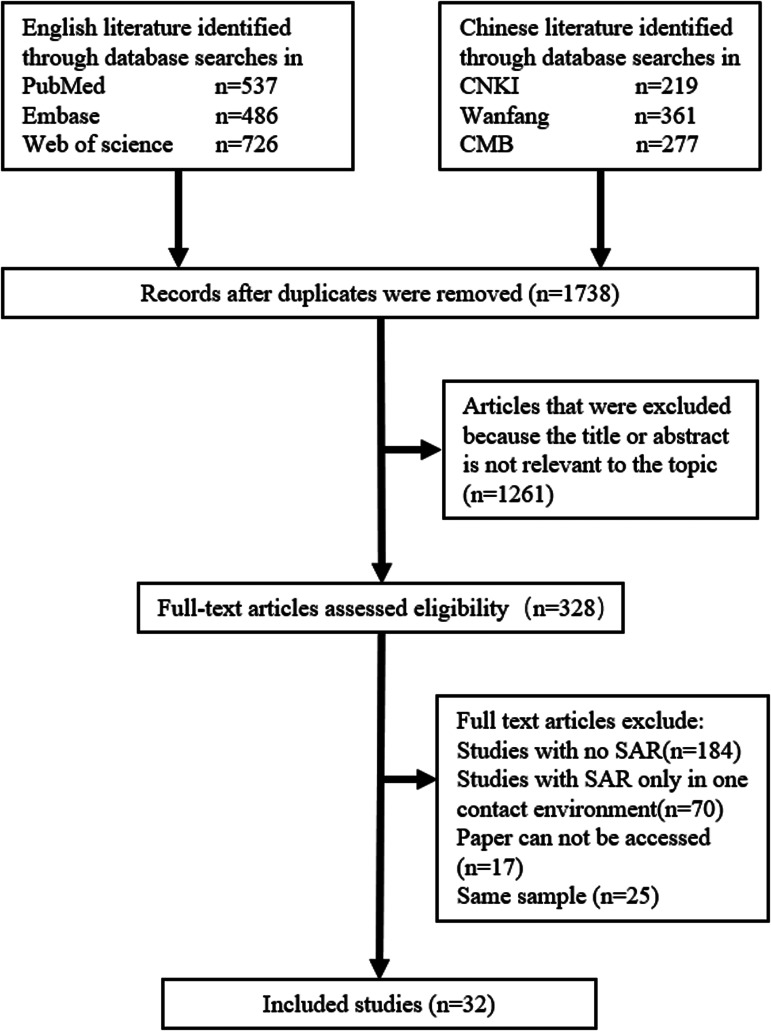
Study selection process.

A total of 2606 citations were retrieved from the literature search, including 857 citations from Chinese databases and 1749 from English databases. Among them, 1738 citations remained after removing duplicates. After screening the titles and abstracts, 1261 studies were excluded. The full texts of the remaining 328 studies were obtained and read. Finally, 296 studies were excluded by reading full text, and 32 studies were included (Fig. 1).

The characteristics of the included studies are presented in Supplementary Table S2. After removing types of contact environments that could not be classified, 32 studies involved 68 260 close contacts were included in the analysis, in which 2406 were infected. The research period of included studies was between January 2020 and March 2021. Among them, 21 studies were from mainland China, 2 were from the United States, 2 were from South Korea and the remaining 9 studies were from Australia, Singapore, Japan, Yemen, Rwanda, India and Taiwan, China, respectively.

### Risk of bias in included studies

Among them, three studies reported the SARs of seven types of contact environments, 13 studies reported the SARs of three types of contact environments. Four studies have not conducted further tracking and PCR testing of all identified close contacts and were considered as low quality. The results of the study quality evaluation are presented in Supplementary File S4.

In 21 pairs of pairwise comparisons, 13 pairs of *I*^2^ values exceed 50%. Heterogeneity of the pairwise comparisons between households and other contact environments was the highest (the values of *I*^2^ were around 85%). The details of heterogeneity are presented in Supplementary Table S5. For all analyses, the Egger's test showed that there was no publication bias (*P* > 0.1). The results of the Egger's test and the funnel plots are presented in Supplementary File S9.

### Outcomes of NMA for all included studies

**Fig. 2.**
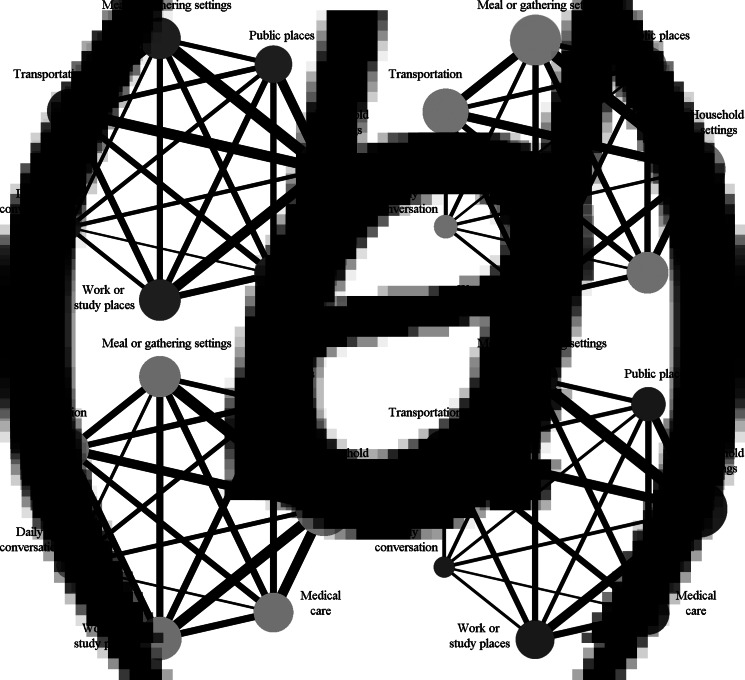
NMA of SAR in seven contact environments. *Note:* (A) All studies; (B) subgroup in mainland China, (C) studies without small sample sizes and (D) studies without low quality. Width of the line is directly proportional to the number of included studies. Size of the node is proportional to the sample size.

Figure 2 shows the network geometry of eligible comparisons for SARs of the seven contact environments, with each contact environment represented by a node. The line between the nodes indicated evidence of a direct comparison between the two contact environments. All contact environments were included in the direct network comparison. Due to the result of the consistency test (*P* = 0.0022), the inconsistency model was applied for fitting the data. The results of side-splitting showed that the results of direct and indirect comparisons were consistent in each pairwise comparison (Supplementary File S6).

Table 2 shows the RRs of the NMA between the seven contact environments. The SAR of meal or gathering settings was nearly 10 times higher than that of transportation (RR 10.55, 95% confidence interval (CI) 1.43–77.85), work or study places (RR 11.68, 95% CI 1.58–86.61) and medical care (RR 10.15, 95% CI 1.40–73.38). As shown in Table 1, the SARs of the SUCRA ranked from the highest to lowest were: household settings (95.3%), meal or gathering settings (81.4%), public places (58.9%), daily conversation (50.1%), transportation (30.8%), medical care (18.2%) and work or study places (15.3%). Household SAR of SUCRA ranked the highest, which means household SAR had the greatest probability of having the highest SAR among all contact environments. But, due to the wide ranges of CIs, there were no statistically significant differences between the SAR of the household and other contact environments.

**Table 1.** Meta-analysis of SARs in different contact environments

	Group	Number of studies	Sample size	NMA		Traditional meta-analysis			
				SUCRA	Rank	SAR (95% CI)	*I*^2^ (%)	*Q* test	*P*^a^
All studies	Household settings	29	15 034	95.3	1	11.7% (9.7–13.8)	96.80	875.69	<0.001
	Meal or gathering	19	8633	81.4	2	6.2% (4.6–7.9)	93.40	273.85	<0.001
	Public places	15	17 094	58.9	3	1.8% (1.2–2.4)	81.50	75.71	<0.001
	Daily conversation	8	2919	50.1	4	5.0% (1.9–8.0)	94.10	119.35	<0.001
	Transportation	18	9115	30.8	5	0.8% (0.4–1.2)	72.00	60.77	<0.001
	Medical care	15	4866	18.2	6	0.7% (0.4–1.1)	34.50	21.36	0.093
	Work or study places	16	5888	15.3	7	3.0% (1.9–4.1)	88.90	135.71	<0.001
Studies from mainland China	Household settings	19	9065	99.9	1	13.9% (11.1–16.8)	95.40	391.92	<0.001
	Meal or gathering	17	8371	79.8	2	6.6% (4.8–8.4)	94.10	271.62	<0.001
	Public places	10	5293	48.3	3	1.9% (1.0–2.7)	80.5	46.16	<0.001
	Daily conversation	5	590	47.9	4	2.9% (−0.3 to 6.1)	61.4	10.35	0.035
	Work or study places	9	2208	40.3	5	1.5% (0.6–2.5)	69.2	26.01	0.001
	Medical care	11	3998	22.8	6	0.8% (0.4–1.1)	23.40	13.06	0.221
	Transportation	13	8480	10.9	7	0.7% (0.3–1.2)	77.60	53.50	<0.001
Studies without small sample sizes	Household settings	25	14 979	99.8	1	11.6% (9.5–13.7)	97.2	859.08	<0.001
	Meal or gathering	16	8565	79.7	2	5.9% (4.3–7.6)	94.3	265.38	<0.001
	Daily conversation	7	2900	59.4	3	4.6 (1.5–7.6)	94.8	115.17	<0.001
	Work or study places	15	5886	52.0	4	3.0% (1.9–4.0)	89.5	132.83	<0.001
	Public places	15	17 094	35.1	5	1.8% (1.2–2.4)	81.5	75.71	<0.001
	Medical care	14	4849	21.1	6	0.7% (0.4–1.0)	30.5	18.70	0.133
	Transportation	13	9020	2.9	7	0.6% (0.3–0.9)	63.7	33.06	0.001
Studies without low-quality	Household settings	26	14 304	92.6	1	12.0% (9.8–14.2)	97.0	830.77	<0.001
	Meal or gathering	19	8633	77.7	2	6.2% (4.6–7.9)	93.4	273.85	<0.001
	Public places	12	7623	54.7	3	1.9% (1.1–2.6)	81.4	59.07	<0.001
	Work or study places	13	4646	46.6	4	1.5% (0.8–2.3)	70.7	40.92	<0.001
	Daily conversation	6	1624	45.5	5	5.6% (0.0–11.3)	95.7	114.98	<0.001
	Transportation	15	8504	18.3	6	0.7% (0.3–1.2)	75.6	57.48	0.221
	Medical care	15	4866	14.7	7	0.7% (0.4–1.1)	34.5	21.36	0.093

SUCRA, the surface under the cumulative ranking curve; SAR, secondary attack rates.

a *P* for *Q* test.

For the results of the traditional meta-analysis, the pooled SAR of each contact environment is listed in Table 1. The pooled SARs was the highest for household SAR as 11.7% (95% CI 9.7%–13.8%), and the lowest one was medical care SAR as 0.7% (95% CI 0.4%–1.1%).

### Outcomes of NMA for studies from mainland China

The network comparisons for SARs in the subgroup of mainland China are also shown in Figure 2. After excluding the 11 studies from regions other than mainland China, the data met the consistency assumption (*P* = 0.1401), and the consistency model was used for fitting.

For the 21 studies conducted in mainland China, the number of RRs of pairwise comparisons with statistical significance increased from three to eight. The SAR of households compared with other environments were all significantly higher (Table 2), in which RRs varied from 2.48 (95% CI 1.35–4.57) for meal or gathering to 9.71 (95% CI 4.70–20.05) for transportation. Besides, the SAR of meal or gathering settings was nearly three times higher than that of transportation and medical care (RR 3.91, 95% CI 1.85–8.27; RR 3.13, 95% CI 1.34–7.29). Compared with the SUCRA for all 32 studies, the relative rankings of SUCRA only exchanged between work or study places and transportation (Table 1).

**Table 2.** Pairwise comparisons of SARs for seven contact environments

**A**						
**Hou**	**5.01** (2.30–10.91)*	**2.48** (1.35–4.57)*	**9.71** (4.70–20.05)*	**4.93** (1.59–15.25)*	**5.73** (2.49–13.20)*	**7.78** (3.49–17.31)*
7.83 (0.98–62.26)	**Pub**	0.50 (0.22–1.14)	1.94 (0.76–4.92)	0.98 (0.28–3.47)	1.14 (0.42–3.09)	1.55 (0.61–3.94)
0.29 (0.03–2.43)	0.46 (0.11–1.86)	**Mea**	**3.91** (1.85–8.27)*	1.98 (0.64–6.15)	2.31 (0.97–5.49)	**3.13** (1.34–7.29)*
0.51 (0.05–5.54)	4.88 (0.64–37.07)	**10.55** (1.43–77.85)*	**Tra**	0.51 (0.16–1.64)	0.59 (0.23–1.52)	0.80 (0.32–2.01)
2.29 (0.21–25.49)	1.56 (0.21–11.43)	3.37 (0.47–23.99)	0.32 (0.06–1.70)	**Dai**	1.16 (0.33–4.09)	1.58 (0.45–5.56)
1.44 (0.14–15.04)	5.40 (0.71–41.17)	**11.68** (1.58–86.61)*	1.11 (0.19–6.39)	3.47 (0.63–19.23)	**Wor**	1.36 (0.50–3.68)
8.60 (0.64–116.04)	4.69 (0.63–34.92)	**10.15** (1.40–73.38)*	0.96 (0.18–5.24)	3.01 (0.58–15.70)	0.87 (0.15–4.90)	**Med**
**B**						
**Hou**	**5.13** (2.74–9.60)*	**2.01** (1.12–3.60)*	**11.04** (5.53–22.04)*	**3.21** (1.34–7.67)*	**3.77** (2.00–7.07)*	**6.77** (3.35–13.67)*
7.86 (0.97–64.06)	**Pub**	**0.39** (0.19–0.81)*	2.15 (0.96–4.81)	0.62 (0.25–1.57)	0.73 (0.35–1.54)	1.32 (0.59–2.94)
0.29 (0.03–2.52)	0.46 (0.11–1.90)	**Mea**	**5.50** (2.58–11.72)*	1.60 (0.62–4.10)	1.88 (0.91–3.87)	**3.37** (1.55–7.33)*
2.29 (0.20–26.40)	4.90 (0.63–38.12)	**10.59** (1.40–79.97)*	**Tra**	**0.29** (0.11–0.77)*	**0.34** (0.15–0.76)*	0.61 (0.26–1.44)
1.44 (0.13–15.59)	1.56 (0.21–11.75)	3.38 (0.46–24.65)	0.32 (0.06–1.73)	**Dai**	1.17 (0.46–3.00)	2.11 (0.76–5.86)
0.18 (0.02–2.01)	5.40 (0.69–42.11)	**11.67** (1.54–88.52)*	1.10 (0.19–6.43)	3.45 (0.62–19.39)	**Wor**	1.80 (0.80–4.06)
3.70 (0.40–34.63)	4.70 (0.62–35.82)	**10.16** (1.37–75.22)*	0.96 (0.17–5.30)	3.01 (0.57–15.88)	0.87 (0.15–4.96)	**Med**

Hou, household settings; Pub, public places; Mea, meal or gathering settings; Tra, transportation; Dai, daily conversation; Wor, work or study places; Med, medical care.

(A) The lower left corner shows the pairwise comparison results of all 32 studies, and the upper right corner shows the results of 21 mainland China. (B) The lower left corner shows the pairwise comparison results of studies without small sample sizes, and the upper right corner shows the results of studies without low quality.

The effect size represents the relative risk (RR, 95% CI) of the SAR of the contact environments on the left relative to the right. Bold and * indicate that the result is statistically significant.

### Sensitivity analysis

Sensitivity analysis was conducted by excluding small sample studies (<100) and studies with low quality, respectively.

After removing the small sample studies, the *P*-value of the consistency test changed to 0.0759 and the consistency model was used for fitting. The results were quite different compared with the results of 32 studies, and there were 11 RRs of pairwise comparison showing significant differences. The RRs of household SAR compared with others were all significantly beyond 1, which was similar to the results of subgroup (Table 2). Among them, household SAR was 2.01 times that of meal or gathering (95% CI 1.12–3.60) and 11.04 times that of transportation (RR 11.04, 95% CI 5.53–22.04). Moreover, meal or gathering SAR was 2.56 times that of public places (RR 2.56, 95% CI 1.24–5.29), 3.37 times that of medical care (RR 3.37, 95% CI 1.55–7.33) and 5.50 times that of transportation (RR 5.50, 95% CI 2.58–11.72). When taking transportation as the reference group, the SAR of daily conversation and work or study places were close to three times that of transportation. In addition, the SUCRA rankings also have changed (Table 1). The daily conversation and the work or study places rose to the 3rd (59.4%) and the 4th (52%), and the public places and the transportation dropped to the 5th (35.1%) and the 7th (2.9%).

After excluding a low-quality study, the 4th to 7th SUCRA rankings changed, and the ranking of the work or study places rose to the 4th. However, the point estimations of the RRs were similar with the results of overall studies (Table 2).

## Discussion

### Main findings

This systematic review included currently the largest number of eligible observational studies involving 68 260 participants of SAR of COVID-19 in varied contact environments. The SARs of seven types of contact environments were compared and the pairwise RRs of them were estimated using the NMA approach for the first time. For all studies, the SAR of meal or gathering was significantly higher than the other three contact environments: Mea *vs.* Tra (RR 10.55, 95% CI 1.43–77.85), Mea *vs.* Wor (RR 11.68, 95% CI 1.58–86.61) and Mea *vs.* Med (RR 10.15, 95% CI 1.40–73.38). The SUCRA rankings of SARs from the highest to the lowest were household, meal or gathering, public places, daily conversation, transportation, medical care and work or study places. Although the SAR of the household settings was higher than other environments, their RRs were not statistically significant.

Our study suggested that the estimates of RRs from mainland China and studies without small sample sizes were more stable, supported by the significant RRs with narrower 95% CIs in NMA and larger *P*-value (>0.05) in consistency test. After excluding 11 non-mainland China studies and five studies with small sample sizes, the statistically significant RRs increased to 8 and 11, respectively. And the RRs between the household SARs and all other environments SARs became statistically significant in both analyses. Besides the household and meal or gathering settings, the SUCRA ranking of other contact environments also changed. These changes were possibly caused by the changes in SARs in diverse contact environments and the removing of small-study effect. The results of the subgroup analysis and sensitivity analysis showed that the SARs are significantly higher among multiple contact environments, especially for household and meal or gathering settings. Therefore, stratified personal protection and public health measures need to be in place accordingly, so as close contacts categorising and management.

Findings in our study could be explained by the characteristics of transmission, and were in line with previous studies. SARS-CoV-2 is mainly spread through respiratory droplets in face-to-face contact [2]. Wearing a mask and keeping social distance can effectively reduce the possibility of transmission [2]. People living together spend more time together and have a closer social distance, which may lead to higher SAR [9, 25]. In healthcare environments, people use more protective measures, such as masks and disinfection, which reduces the probability of infection [3].

There were some potential confounders in our study, which may lead to inaccurate results, especially for the analysis of the overall studies. Multiple factors might influence the results, such as the population of the country, criteria for determining close contacts and lockdown status [7]. These regional factors could increase the heterogeneity of our study. Also, there were some factors considered as the confounder factors of SARs, such as asymptomatic detection rates and the status of herd immunity [6, 10, 25]. Some reviews suggested that the SAR of symptomatic cases was higher than asymptomatic cases because symptomatic cases release more viruses during the onset of symptoms, making close contacts more susceptible to infection [6, 10, 25]. Since the asymptomatic detection rates were almost equal in diverse contact environments in each study, the effects of the factor were partly offset and attenuated when calculating the RRs of SARs. For herd immunity, although whether the antibodies were good to prevent re-infection still needed to be confirmed, the status of herd immunity could be a potential confounder for SARs [26]. But, when the status of herd immunity was similarly in the compared contact environments, the confounder effects may also be offset and attenuated.

### The differences in regions

In our study, the results in mainland China were different from the overall studies. There may be several reasons. Most of the studies included in this study were from mainland China, which was possibly because mainland China has more detailed and unified management and classification of close contacts than many other countries. After excluding 11 studies from non-mainland China, results became consistent, estimates became significant and more accurate as the 95% CIs narrowed, indicating higher stability and reliability, and SUCRA ranking and the RRs of pairwise comparisons changed. These differences may be caused by different standards for determining close contacts and management programmes in China and other regions.

There were some studies in other countries that reported close contacts, but their management classification of exposure to the environments varied and often simplified, which mainly focused on household SAR [27–29]. China has conducted strict and detailed epidemiological information surveys of each close contact, and classified the contact environments into seven types according to national guidelines [15]. The World Health Organization's guidance for contact tracing classification was similar to the Chinese guideline, which has six categories including household contacts, closed settings, healthcare settings, professional contacts, public or shared transport and other well-defined settings [30]. The US Centre for Disease Control and Prevention provides risk assessment information and guidelines for close contacts, including school work environment, travel, community and medical care environment [31]. In Taiwan China, the Ministry of Health and Welfare suggested that contact environments be classified into four types, including hospital contacts, airplane contacts, school contacts and others [32]. The health departments of Singapore, South Korea and Australia did not have a clear classification of the settings of close contacts [33–35]. Besides, the detection rate by contact environments between different regions may also influence the results. In China, almost all the close contacts were conducted further tracking and PCR testing, but the situation in other countries was different, such as Australia, India, Rwanda, Japan and United States, which might cause potential attrition bias introduced by the loss of follow-up [36–40].

Meanwhile, in the early stage of the epidemic, the Chinese government conducted strict social isolation measures, which might lead to more contact frequency and opportunities in households than in other environments [41]. This might also be a potential cause of significant results when calculating the RRs between household SAR and other environments in mainland China.

### Sensibility analysis of the study

In our study, the results excluding small sample studies were more stable and credible. More RRs showed significance, and their 95% CIs became much narrower. The risk of household SAR was significantly higher than that of other contact environments. The order of 3rd to 7th in the SUCRA ranking has also changed. Small sample studies may lead to bias because of potential publication bias [42] and instability of effect value of SARs [43]. Compared with large sample studies, the method of correcting zero events in the STATA software network package might cause a larger biased estimate for small sample studies, which may also cause NMA results including small sample studies to be more unstable [12]. Also, the inconsistency model might lead to a wider 95% CI than the consistency model, which may also contribute to the insignificant results [20].

Besides the small-study effect, other biases of studies included in our studies were acceptable. Except for the higher risk of bias in clinical features (no clinical features of follow-up cases reported), which was not the focus of this study, the included studies have a lower risk of bias. Most studies might have a bias in one respect. After excluding low-quality studies, the 4th–7th SUCRA rankings changed, and the ranking of the work or study places group rose to 4th. But, the RRs were similar. Due to the decrease in the number of studies and participants included, the uncertainty of the results has increased and the SUCRA ranking has changed [44, 45]. It indicated the instability of the SUCRA ranking once again, and suggested that a conclusion should not be made on the results of SUCRA only, but with the effect size of the pairwise comparison [44].

### Limitations of the study

This study has some limitations. First, due to the lack of specific quality evaluation tools for retrospective epidemiological studies on infectious diseases, after examining several risks of bias assessment tools, the JBI tool was selected to evaluate the biases, and an adaptive explanation was made for each question according to general principles of epidemiological studies [14]. But, this may still lead to certain errors or inaccuracies of quality evaluation. Therefore, to obtain more rigorous evidence through the NMA, methodological improvement is still needed.

Second, we planned to do subgroup analysis by region, age and other confounder factors. However, there were few studies reporting available demographic characteristics. Some only reported the demographic characteristics of the follow-up cases instead of all the close contacts. Some reported the demographic characteristics of overall close contact rather than that of each contact environment group. Due to the lack of information, we only carried out subgroup analysis by region eventually.

Finally, due to the lack of information on the classification of contact environments, we excluded some studies that could not be included in the seven classifications. Meanwhile, due to the method limitations of the NMA, we only included studies that reported at least two groups of exposure routes; in other words, studies that reported only one exposure route and total SAR were not included in our study, which might miss some information. And, NMA could not estimate the pooled SAR in each contact environment, so traditional meta-analysis was conducted to calculate the pooled SARs as Supplementary results.

## Conclusion

Overall, household SAR has the probability of being the highest SAR among SARs of all contact environments, followed by SARs of meal or gathering, public places, daily conversation, transportation, medical care and work or study places. Household SARs were significantly higher than other environments supported by more accurate results using studies from China and sensitive analysis without small sample studies (<100). This supported the necessity of classifying and managing close contacts using stratified strategies in the COVID-19 epidemic.

## Data Availability

All the data were from the published studies. The dataset is available from the corresponding author by reasonable request (Jiayuan Li; E-mail: lijiayuan@scu.edu.cn).

## Supplementary material

For supplementary material accompanying this paper visit http://dx.doi.org/10.1017/S0950268821002223. click here to view supplementary material

## References

[1] Listings of WHO's response to COVID-19. Available at https://www.who.int/news/item/29-06-2020-covidtimeline (Accessed 29 June 2020).

[2] Wiersinga WJ (2020) Pathophysiology, transmission, diagnosis, and treatment of coronavirus disease 2019 (COVID-19): a review. JAMA 324, 782–793. Published online: 11 July 2020. 32648899 10.1001/jama.2020.12839

[3] Chu DK (2020) Physical distancing, face masks, and eye protection to prevent person-to-person transmission of SARS-CoV-2 and COVID-19: a systematic review and meta-analysis. Lancet (London, England) 395, 1973–1987. Published online: 5 June 2020. 10.1016/S0140-6736(20)31142-9 PMC7263814 32497510

[4] Hui-hui Z (2020) Epidemic characteristics of close contacts of coronavirus disease 2019 in Xi'an. Journal of Xi'an Jiaotong University (Medical Sciences) 41, 502–505.

[5] Hui-Jun L (2020) Treatment and analysis of a clustering pneumonia caused by 2019-nCoV in Wuzhi county. Henan Journal of Preventive Medicine 31, 573–576.

[6] Madewell ZJ (2020) Household transmission of SARS-CoV-2: a systematic review and meta-analysis. JAMA Network Open 3, e2031756. Published online: 15 December 2020. 33315116 10.1001/jamanetworkopen.2020.31756 PMC7737089

[7] Shah K (2020) Secondary attack rate of COVID-19 in household contacts: a systematic review. QJM: Monthly Journal of the Association of Physicians 113, 841–850. Published online: 30 July 2020. 32726452 10.1093/qjmed/hcaa232 PMC7454929

[8] Fung HF (2020) The household secondary attack rate of SARS-CoV-2: a rapid review. Clinical Infectious Diseases 73, S138–S145. 10.1093/cid/ciaa1558 PMC7665336 33045075

[9] Tian T and Huo X (2020) Secondary attack rates of COVID-19 in diverse contact settings, a meta-analysis. The Journal of Infection in Developing Countries 14, 1361–1367. 33378276 10.3855/jidc.13256

[10] Thompson HA (2021) SARS-CoV-2 setting-specific transmission rates: a systematic review and meta-analysis. Clinical Infectious Diseases 73, e754–e764. 33560412 10.1093/cid/ciab100 PMC7929012

[11] Andrea C (2013) Conceptual and technical challenges in network meta-analysis. Annals of Internal Medicine 159, 130–137. 23856683 10.7326/0003-4819-159-2-201307160-00008

[12] Shim S (2017) Network meta-analysis: application and practice using Stata. Epidemiology and Health 39, e2017047. Published online: 3 November 2017. 29092392 10.4178/epih.e2017047 PMC5733388

[13] Hutton B (2014) Checking whether there is an increased risk of post-transplant lymphoproliferative disorder and other cancers with specific modern immunosuppression regimens in renal transplantation: protocol for a network meta-analysis of randomized and observational studies. Systematic Reviews 3, 16. Published online: 25 February 2014. 24559430 10.1186/2046-4053-3-16 PMC3936935

[14] The Joanna Briggs Institute. Joanna Briggs institute reviewers' manual. In: Institute TJB, editor. Australia 2016.

[15] The Guideline of COVID-19 (2020) Control and Prevention-Close Contact Management Procedure (7th ed).

[16] Lin Z (2020) Incidence analysis of 1403 close contacts of corona virus disease 2019 patients in different contact modes. Journal of Shandong University (Health Sciences) 58, 58–61, Published online: 4 August 2020.

[17] Liu Y (2020) Secondary attack rate and superspreading events for SARS-CoV-2. Lancet (London, England) 395, e47. Published online: 3 March 2020. 10.1016/S0140-6736(20)30462-1 PMC7158947 32113505

[18] Dias S and Caldwell DM (2019) Network meta-analysis explained. Archives of Disease in Childhood-Fetal and Neonatal Edition 104, F8–F12. Published online: 15 November 2018. 30425115 10.1136/archdischild-2018-315224 PMC6761999

[19] Watt J (2019) Research techniques made simple: network meta-analysis. Journal of Investigative Dermatology 139, 4–12.e11. Published online: 24 December 2018. 10.1016/j.jid.2018.10.028 30579427

[20] Higgins JP (2012) Consistency and inconsistency in network meta-analysis: concepts and models for multi-arm studies. Research Synthesis Methods 3, 98–110. Published online: 1 June 2012. 26062084 10.1002/jrsm.1044 PMC4433772

[21] Yu-Kang T (2016) Node-splitting generalized linear mixed models for evaluation of inconsistency in network meta-analysis. Value in Health 19, 957–963. Published online: 19 December 2016. 27987646 10.1016/j.jval.2016.07.005

[22] Bo Z Meta-Analysis of Rates and Software Implementation. doi: 10.7507/1672-2531.20140166

[23] Dawson DV (2016) Understanding and evaluating meta-analysis. Journal of the American Dental Association 147, 264–270. Published online: 27 December 2015. 26705602 10.1016/j.adaj.2015.10.023

[24] Harris RJ (2008) Metan: fixed- and random-effects meta-analysis. The Stata Journal 8, 3–28.

[25] Koh WC (2020) What do we know about SARS-CoV-2 transmission? A systematic review and meta-analysis of the secondary attack rate and associated risk factors. PLoS One 15, e0240205. 33031427 10.1371/journal.pone.0240205 PMC7544065

[26] Poonia B and Kottilil S. (2020) Immune correlates of COVID-19 control. Frontiers in Immunology 11, 569611. Published online: 3 November 2020. 33133083 10.3389/fimmu.2020.569611 PMC7550526

[27] Ng OT (2020) SARS-CoV-2 seroprevalence and transmission risk factors among high-risk close contacts: a retrospective cohort study. Lancet Infectious Diseases 21, 333–343. 33152271 10.1016/S1473-3099(20)30833-1 PMC7831879

[28] Li W (2020) Characteristics of household transmission of COVID-19. Clinical Infectious Diseases 71, 1943–1946. Published online: 18 April 2020. 32301964 10.1093/cid/ciaa450 PMC7184465

[29] Wilkinson K (2021) Secondary attack rate of COVID-19 in household contacts in the Winnipeg Health Region, Canada. Canadian Journal of Public Health Revue Canadienne de Santé Publique 112, 12–16. Published online: 19 November 2020. 33205377 10.17269/s41997-020-00451-x PMC7671665

[30] Organization WH (2021) Contact tracing in the context of COVID-19 interim guidance.

[31] Centers for Disease Control and Prevention. Available at https://www.cdc.gov/coronavirus/2019-ncov/index.html (Accessed 17 August 2021).

[32] Ministry of Health and Welfare. Guidelines for investigation and contact tracing of severe special infectious pneumonia. Available at https://www.cdc.gov.tw/File/Get/B0h3BBa-bXGwwBwkxoUb1g (Accessed 22 January 2020).

[33] What is home quarantine order？. Available at https://www.moh.gov.sg/covid-19/general/resources (Accessed 17 August 2021).

[34] Australian Health Sector Emergency Response Plan for Novel Coronavirus (COVID-19). Available at https://www.health.gov.au/sites/default/files/documents/2020/02/australian-health-sector-emergency-response-plan-for-novel-coronavirus-covid-19_2.pdf (Accessed 7 February 2020).

[35] Zastrow M. How South Korea prevented a coronavirus disaster – and why the battle isn't over. Available at https://www.nationalgeographic.com/science/article/how-south-korea-prevented-coronavirus-disaster-why-battle-is-not-over (Accessed 12 May 2020).

[36] Draper AD (2020) The first 2 months of COVID-19 contact tracing in the northern territory of Australia, March–April 2020. Communicable Diseases Intelligence (2018) 44. doi: 10.33321/cdi.2020.44.53. 32615916

[37] Burke RM (2020) Enhanced contact investigations for nine early travel-related cases of SARS-CoV-2 in the United States. PLoS One 15, e0238342. Published online: 2020/09/03. 32877446 10.1371/journal.pone.0238342 PMC7467265

[38] Akaishi T (2021) COVID-19 transmission in group living environments and households. Scientific Reports 11, 11616. Published online: 4 June 2021. 34079047 10.1038/s41598-021-91220-4 PMC8172911

[39] Semakula M (2021) The secondary transmission pattern of COVID-19 based on contact tracing in Rwanda. BMJ Global Health 6, e004885. 10.1136/bmjgh-2020-004885 PMC8189754 34103325

[40] Sundar V and Bhaskar E (2021) Low secondary transmission rates of SARS-CoV-2 infection among contacts of construction laborers at open air environment. Germs 11, 128–131. Published online: 27 April 2021. 33898351 10.18683/germs.2021.1250 PMC8057850

[41] Wang J and Wang Z (2020) Strengths, Weaknesses, Opportunities and Threats (SWOT) analysis of China's prevention and control strategy for the COVID-19 epidemic. International Journal of Environmental Research and Public Health 17, 2235. 10.3390/ijerph17072235 PMC7178153 32225019

[42] Ioannidis JP (1998) Issues in comparisons between meta-analyses and large trials. JAMA 279, 1089–1093. Published online: 18 April 1998. 9546568 10.1001/jama.279.14.1089

[43] Pereira TV (2012) Empirical evaluation of very large treatment effects of medical interventions. JAMA 308, 1676–1684. Published online: 25 October 2012. 23093165 10.1001/jama.2012.13444

[44] Wang Z and Carter RE (2018) Ranking of the most effective treatments for cardiovascular disease using SUCRA: is it as sweet as it appears? European Journal of Preventive Cardiology 25, 842–843. Published online: 24 March 2018. 29569939 10.1177/2047487318767199

[45] Jansen JP (2014) Indirect treatment comparison/network meta-analysis study questionnaire to assess relevance and credibility to inform health care decision making: an ISPOR-AMCP-NPC good practice task force report. Value in Health 17, 157–173. 24636374 10.1016/j.jval.2014.01.004

