# Supplementary material for: A network meta-analysis of secondary attack rates of COVID-19 in different contact environments
Source: Epidemiol Infect. 2021 Oct 5;149:e219. doi: 10.1017/S0950268821002223 (PMC8523971; doi:10.1017/S0950268821002223)

**Supplementary Appendix**

**1. Supplementary Table 1. Strategies of search**

**2. Supplementary File 2. Included studies**

**2.1 Supplementary Table 2. Characteristics of included studies**

**2.2 Reference list of included studies**

**3. Supplementary Table 3. The classification details of the seven contact environments**

**4. Supplementary File 4. Literature quality evaluation**

**4.1 Supplementary Figure 1. quality assessment of included studies**

**4.2 Supplementary table 4. quality assessment of each study**

**5. Supplementary table 5. Estimation of heterogeneity in pairwise comparisons**

**6. Supplementary File 6. Estimation of inconsistency**

**6.1 Supplementary table 6. Estimation of inconsistency model**

**6.2 The side-splitting (node-splitting) method and Loop-specific approach**

**7. Supplementary File 7. SUCRA and cumulative probability plots**

**8. Supplementary File 8. Forest plot of the effect estimates for pairwise comparison**

**9. Supplementary File 9. Funnel plots and Egger’s test**

**1. Supplementary Table 1.** **Strategies of search**

| **Database** | **Specific strategy** | **Number of studies** |
| --- | --- | --- |
| CNKI^a^ | (Title=新冠 + 冠状病毒 + COVID + nCoV) AND (Title/Abstract/Keywords=密切接触 + 聚集性疫情 + 续发 + 发病 ) AND (Title/Abstract/Keywords =接触途径 + 接触方式 + 接触场所 + 传播途径 + 传播方式 + 传播场所) | 219 |
| Wanfang | Title: (新冠 OR 冠状病毒 OR COVID OR nCoV)* Topic: (密切接触 OR 聚集性疫情 OR 续发 OR 发病)* Topic: (接触途径 OR 接触方式 OR 接触场所 OR 传播途径 OR 传播方式 OR 传播场所) | 361 |
| CBM^b^ | ( "新冠"[Common field: smart] OR "冠状病毒"[Common field: smart] OR "COVID"[Common field: smart] OR "nCoV"[Common field: smart]) AND( "密切接触"[Common field: smart] OR "聚集性疫情"[Common field: smart] OR "续发"[Common field: smart] OR "发病"[Common field: smart]) AND( "接触途径"[Common field: smart] OR "接触方式"[Common field: smart] OR "接触场所"[Common field: smart] OR "传播途径"[Common field: smart] OR "传播方式"[Common field: smart] OR "传播场所"[Common field: smart]) | 277 |
| Web of Science | Web of Science: Title: (novel corona* OR new corona* OR CoV or nCoV OR COVID* OR SARS-CoV-2 OR SARS2) AND Topic: (Close contact* OR family cluster OR secondary attack rate OR SAR) AND Topic: (Transmission OR contact trac*) | 726 |
| Embase | (novel corona* or new corona* or CoV or nCoV or COVID* or SARS-CoV-2 or SARS2).ti. and (Close contact* or family cluster or secondary attack rate or SAR).ab. and (Transmission or contact trac*).ab. | 486 |
| Pubmed | ((novel corona*[Title] OR new corona*[Title] OR CoV[Title] OR nCoV[Title] OR COVID*[Title] OR SARS-CoV-2[Title] OR SARS2[Title]) AND (Close contact*[Title/Abstract] OR family cluster[Title/Abstract] OR secondary attack rate[Title/Abstract] OR SAR[Title/Abstract])) AND (Transmission[Title/Abstract] OR contact trac*[Title/Abstract]) | 537 |

^a^ Chinese literature databases of China National Knowledge Infrastructure

^b^ China Biology Medicine disc

**2.** **Supplementary File 2. Included studies**

**2.1 Supplementary Table 2. Characteristics of included studies**

| First author | Publication date | Location | Study Period | Close contacts | Follow-up cases | Age (mean) | Male% | Study type | Types of contact environments |
| --- | --- | --- | --- | --- | --- | --- | --- | --- | --- |
| Draper | Jul-20 | Australia | 2020.3.1-2020.4.30 | 423 | 4 | - | - | Retrospective epidemiological research | ADG |
| Jeong | Mar-20 | Korea | 2020.1.24-2020.3.10 | 390 | 9 | 45^a^ | 45.0 | Retrospective epidemiological research | AFG |
| Han | Jul-20 | Korea | 2020.3.28-2020.4.8 | 36 | 7 | 2~73^b,c^ | 30.0^c^ | Retrospective epidemiological research | ACD |
| Burke | Sep-20 | America | 2020.1-2020.2 | 256 | 2 | 18~44^b^ | 37.0 | Cluster epidemic report | ABG |
| Ng | Nov-20 | Singapore | 2020.1.23-2020.4.3 | 7518 | 180 | 10~53^b^ | 47.6 | Retrospective epidemiological research | AF |
| Huang | Jun-20 | Taiwan, China | 2020.1.21-2020.4.8 | 2899 | 29 | - | - | Retrospective epidemiological research | ACFG |
| Wang | Jul-20 | Hefei, Anhui, China | 2020.1.21-2020.2.29 | 847 | 58 | 38.4 | 48.5 | Retrospective epidemiological research | ABCDEFG |
| Jia | Oct-20 | Beijing, China | 2020.2-2020.4 | 1169 | 26 | 27.4^a^ | 45.6 | Retrospective epidemiological research | ACDFG |
| Chen | May-20 | Fujian, China | 2020.1.26-2020.3.4 | 2530 | 111 | 49.5^a, c^ | 52.4^c^ | Retrospective epidemiological research | ABFG |
| Luo | Aug-20 | Guangzhou, Guangdong, China | 2020.1.13-2020.3.6 | 3387 | 124 | 38 | 52.8 | Retrospective epidemiological research | ABDG |
| Bi | May-20 | Shenzhen, Guangdong, China | 2020.1.14-2020.2.12 | 1711 | 156 | 46.3^a^ | 48.0 | Retrospective epidemiological research | ACD |
| Liu | Jul-20 | Nanning, Guangxi, China | 2020.1-2020.2 | 293 | 35 | 44^a, c^ | 47.2^c^ | Retrospective epidemiological research | ACF |
| Lei | Apr-20 | Guizhou, China | 2020.1.21-2020.3.10 | 560 | 69 | 37.5^a, c^ | 64.3^c^ | Retrospective epidemiological research | CG |
| Li | Aug-20 | Wuzhi, Henan, China | 2020.2.14 | 64 | 15 | - | - | Cluster epidemic report | ACD |
| Bao | May-20 | Nanjing, Jiangsu, China | 2020.1-2020.2 | 1812 | 56 | 1~91^b,c^ | 64.3^c^ | Cluster epidemic report | ABC |
| Xv | May-20 | Yangzhou, Jiangsu, China | 2019.12.31-2020.2.29 | 128 | 16 | 43.6 | 48.0 | Retrospective epidemiological research | AC |
| Deng | Sep-20 | Nanchang, Jiangxi, China | 2020.1.21-2020.3.5 | 520 | 42 | - | - | Cluster epidemic report | ABCDFG |
| Chen | Mar-20 | Pingxiang, Jiangxi, China | 2020.1.28-2020.2.9 | 50 | 3 | 57^a, c^ | 0.0^c^ | Cluster epidemic report | AD |
| Zhang | Apr-20 | Liaoning, China | 2020.1.22-2020.2.29 | 1896 | 67 | 40.3^a^ | 49.0 | Retrospective epidemiological research | ACDEF |
| Zhou | Apr-20 | Jinan, Shandong, China | 2020.1.24-2020.2.29 | 1543 | 25 | 57^a^ | 36.5 | Retrospective epidemiological research | ABCDEFG |
| Zhang | Mar-20 | Xi'an, Shaanxi, China | 2020.1.22-2020.2.28 | 4899 | 102 | 38.4 | 48.9 | Retrospective epidemiological research | ABCDFG |
| Miao | Apr-20 | Sichuan, China | 2019.12.31-2020.6.17 | 12299 | 267 | 39^a^ | 51.7 | Retrospective epidemiological research | ACDG |
| Wu | Jun-20 | Hangzhou, Zhejiang, China | 2020.1.23-2020.2.28 | 1752 | 65 | - | 48.9 | Retrospective epidemiological research | ABG |
| Shen | Jul-20 | Jiaxing, Zhejiang, China | 2020.1-2020.2 | 480 | 5 | - | - | Cluster epidemic report | AC |
| Chen | May-20 | Ningbo, Zhejiang, China | 2020.1.21-2020.3.6 | 2050 | 100 | - | - | Retrospective epidemiological research | ABCDEFG |
| Wang | Mar-20 | Pingyang, Zhejiang, China | 2020.1.18-2020.2.6 | 52 | 10 | 48^a, c^ | - | Cluster epidemic report | CDE |
| Zhao | Jun-20 | Chongqing, China | 2019.12.31-2020.4.30 | 1166 | 121 | - | 53.1 | Retrospective epidemiological research | ABC |
| Akaishi | Jun-21 | Sendai, Miyagi, Japan | 2020.7-2021.3 | 4550 | 355 | - | 51.2 | Retrospective epidemiological research | ABE |
| Al-Sakkaf | Apr-21 | Sana’a, Yemen | 2020.4 | 54 | 4 | 27.3^a^ | 61.0 | Cluster epidemic report | ADFG |
| Gettings | Apr-21 | Atlanta, GA, USA | 2020.12.1-2021.1.22 | 672 | 59 | - | - | Retrospective epidemiological research | BDEF |
| Semakula | Jun-21 | Rwanda | 2020.3.14-2020.7.20 | 11809 | 209 | 27.6^a^ | 51.4 | Retrospective epidemiological research | ABDEF |
| Sundar | Mar-21 | India | 2020.4.1-2020.4.20 | 445 | 75 | 34.2 | 82.9 | Cluster epidemic report | ABF |

^a^ median, ^b^ range of age, ^c^ information of follow-up cases

A=household settings. B= public places (entertainment, shopping, socializing, etc.). C=meal or gathering settings. D=transportation. E=daily conversation. F=work or study places. G=medical care.

**2.2 Reference list of included studies**

**(1)** Draper AD, *et al*. (2020) The first 2 months of COVID-19 contact tracing in the Northern Territory of Australia, March-April 2020. Communicable diseases intelligence (2018). 44. doi: http://dx.doi.org/10.33321/cdi.2020.44.53.

**(2)** Jeong EK, *et al*. (2020) Coronavirus disease-19: Summary of 2,370 contact investigations of the first 30 cases in the Republic of Korea. Osong Public Health and Research Perspectives. 11: 81-84. doi: http://dx.doi.org/10.24171/j.phrp.2020.11.2.04.

**(3)** Han T. (2020) Outbreak investigation: transmission of COVID-19 started from a spa facility in a local community in Korea. Epidemiology and Health. 42: e2020056. doi: http://dx.doi.org/10.4178/epih.e2020056.

**(4)** Burke RM, *et al*. (2020) Enhanced contact investigations for nine early travel-related cases of SARS-CoV-2 in the United States. PloS One. 15. doi: 10.1371/journal.pone.0238342.

**(5)** Ng OT, *et al*. (2020) SARS-CoV-2 seroprevalence and transmission risk factors among high-risk close contacts: a retrospective cohort study. Lancet Infectious Diseases. Published online: 6 November 2020. doi: 10.1016/s1473-3099(20)30833-1.

**(6)** Huang YT, *et al*. (2020) Estimation of the secondary attack rate of COVID-19 using proportional meta-analysis of nationwide contact tracing data in Taiwan. Journal of Microbiology, Immunology, and Infection Wei Mian Yu Gan Ran Za Zhi. Published online: 20 June 2020. doi: 10.1016/j.jmii.2020.06.003.

**(7)** Qian W, *et al*. (2020) Incidence of coronavirus disease 2019 among close contacts of confirmed COVID-19 cases in Hefei city. Practical Preventive Medicine. 27: 769-771.

**(8)** Hai-Xian J, *et al*. (2020) Epidemiological characteristics of close contacts of imported COVID-19 cases in Beijing. International Journal of Virology. 27: 367-371.

**(9)** Wu C, *et al*. (2020) Epidemiological characteristics and infection risk factors of people with close contact with coronavirus disease 2019 patients in Fujian Province. Chinese Journal of Disease Control & Prevention. 24: 562-566,585. doi: 10.16462/j.cnki.zhjbkz.2020.05.013.

**(10)** Luo L, *et al*. (2020) Contact Settings and Risk for Transmission in 3410 Close Contacts of Patients With COVID-19 in Guangzhou, China : A Prospective Cohort Study. Annals of Internal Medicine. 173: 879-887. Published online: 14 August 2020. doi: 10.7326/M20-2671.

**(11)** Bi QF, *et al*. (2020) Epidemiology and transmission of COVID-19 in 391 cases and 1286 of their close contacts in Shenzhen, China: a retrospective cohort study. Lancet Infectious Diseases. 20: 911-919. doi: 10.1016/s1473-3099(20)30287-5.

**(12)** Hao-Hui L, *et al*. (2020) Epidemiological characteristics of COVID-19 clusters in Nanning. Preventive Medicine. 32: 674-677+681.

**(13)** Ming-Yu L, *et al*. (2020) Characteristics of cluster epidemic of 2019 novel coronavirus disease in Guizhou province. Chinese Journal of Public Health. 36: 493-497. doi: 10.11847/zgggws1128991.

**(14)** Hui-Jun L, *et al*. (2020) Treatment and analysis of a clustering pneumonia caused by 2019-nCoV in Wuzhi County. Henan Journal of Preventive Medicine. 31: 573-576.

**(15)** Bao C, *et al*. (2020) COVID-19 outbreak following a single patient exposure at an entertainment site: An epidemiological study. Transboundary and Emerging Diseases. doi: http://dx.doi.org/10.1111/tbed.13742.

**(16)** Chun X, *et al*. (2020) Epidemiological characteristics of novel coronavirus pneumonia case close contats in Yangzhou. Jiangsu Journal of Preventive Medicine. 31: 269-270,274. doi: 10.13668/j.issn.1006-9070.2020.03.010.

**(17)** Zhi-Qiang D, *et al*. (2020) Analysis on transmission chain of a cluster epidemic of COVID-19, Nanchang. Chinese Journal of Epidemiology. 41. Published online: 20200418.

**(18)** Feng-Yang C, Li S-X. (2020) Investigation on a cluster of novel coronavirus pneumonia in a certain place in Jiangxi Jiangxi Medical Journal. 55. Published online: 4 August 2020.

**(19)** Rui Z, *et al*. (2020) Infection risk and its influencing factors among close contacts of patients with novel coronavirus disease 2019 in Liaoning province. Chinese Journal of Public Health. 36: 477-480.

**(20)** Lin Z, *et al*. (2020) Incidence analysis of 1403 close contacts of corona virus disease 2019 patients in different contact modes. Journal of Shandong University (Health Sciences). 58: 58-61.

**(21)** Hui-hui Z, *et al*. (2020) Epidemic characteristics of close contacts of coronavirus disease 2019 in Xi'an. Journal of Xi'an Jiaotong University(Medical Sciences). 1-7. Published online: 22 March 2020.

**(22)** Yun-qi M, *et al*. (2021) Infection risk analysis of close contacts of COVID-19 under different exposure conditions in Sichuan Province. Modern Preventive Medicine. 48: 1495-1498.

**(23)** Wu Y*, et al.* (2020) Risk of SARS-CoV-2 infection among contacts of individuals with COVID-19 in Hangzhou, China. Public Health. 185: 57-59. Published online: 22 June 2020. doi: 10.1016/j.puhe.2020.05.016.

**(24)** Shen Y, *et al*. (2020) A Cluster of Novel Coronavirus Disease 2019 Infections Indicating Person-to-Person Transmission Among Casual Contacts From Social Gatherings: An Outbreak Case-Contact Investigation. Open Forum Infect Dis. 7: ofaa231. Published online: 3 July 2020. doi: 10.1093/ofid/ofaa231.

**(25)** Yi C, *et al*. (2020) Epidemiological characteristics of infection in COVID-19 close contacts in Ningbo city. Chinese Journal of Epidemiology. 41: E026-E026. Published online: 29 March 2020.

**(26)** Ke-Shun W, *et al*. (2020) Investigation on a cluster of coronavirus disease 2019 in Pingyang County. Preventive Medicine. 32: 222-225. Published online: 4 August 2020.

**(27)** Han Z, *et al*. (2020) Investigation of transmission chain of a cluster COVID-19 cases. Chinese Journal of Epidemiology. 41: E064-E064. Published online: 16 May 2020.

**(28)** Akaishi T, *et al*. (2021) COVID-19 transmission in group living environments and households. Scientific Reports. 11: 11616. Published online: 4 June 2021. doi: 10.1038/s41598-021-91220-4.

**(29)** Al-Sakkaf E, *et al*. (2021) First COVID-19 cases with high secondary infection among health workers, Sana'a capital, April 2020: Lessons learned and future opportunities. International Journal of Infectious Diseases. Published online: 26 April 2021. doi: 10.1016/j.ijid.2021.04.022.

**(30)** Gettings JR, *et al*. (2021) SARS-CoV-2 transmission in a Georgia school district - United States, December 2020-January 2021. Clinical Infectious Diseases. Published online: 18 April 2021. doi: 10.1093/cid/ciab332.

**(31)** Semakula M, et al. (2021) The secondary transmission pattern of COVID-19 based on contact tracing in Rwanda. BMJ Glob Health. 6. Published online: 10 June 2021. doi: 10.1136/bmjgh-2020-004885.

**(32)** Sundar V, Bhaskar E. (2021) Low secondary transmission rates of SARS-CoV-2 infection among contacts of construction laborers at open air environment. Germs. 11: 128-131. Published online: 27 April 2021. doi: 10.18683/germs.2021.1250.

**3. Supplementary Table 3. The classification details of the seven contact environments**

| group | included situations |
| --- | --- |
| Household settings | Live with SARS-CoV-2 cases.  Expressed in the original research as family members, family gathering, family contact, household contact, household settings, et al. |
| Public places | Stay with SARS-CoV-2 cases in a same public place.  Expressed in the original research as community exposure, entertainment activities, business services, in a same building, in a same activity environment, contact between neighbors, public places, et al. |
| Meal or gathering | Eat with SARS-CoV-2 cases or have face-to-face parties.  Expressed in the original research as: have a meal together, gathering, co-eating, group activities or parties, et al. |
| Transportation | Share transportation with SARS-CoV-2 cases.  Expressed in the original research as in a same cruise, in a same airplane, shared transportation, in a same car, travel, et al. |
| Daily conversation | Have a one-time conversation or stay in the same enclosed space for a short time.  Expressed in the original research as stay in a same room, short-term conversation, daily conversation, et al. |
| Work or study places | Contact SARS-CoV-2 cases in a workplace or school.  Expressed in the original research as school, workplace, professional contacts (other than healthcare settings), et al. |
| Medical care | Contact SARS-CoV-2 cases in healthcare settings.  Expressed in the original research as community healthcare setting, medical staff, hospital, healthcare setting, diagnosis and treatment, nursing (nursing home), medical contact, medical institution, et al. |

**4. Supplementary File 4. Literature quality evaluation**

**4.1 Supplementary Figure 1. quality assessment of included studies**

**Supplementary Figure 1. Risk of bias table of included studies**

*JBI Critical Appraisal Tool for Case Series was used to evaluate the quality of studies. The subjects of the study changed from case series to close contacts and those with subsequent illnesses.

| **4.2 Supplementary Table 4. quality assessment of each study** | | | | | | | | | | |
| --- | --- | --- | --- | --- | --- | --- | --- | --- | --- | --- |
| study | Were there clear criteria for inclusion in the case series? | Was the condition measured in a standard, reliable way for all participants included in the case series? | Were valid methods used for identification of the condition for all participants included in the case series? | Did the case series have consecutive inclusion of participants? | Did the case series have complete inclusion of participants? | Was there clear reporting of the demographics of the participants in the study? | Was there clear reporting of clinical information of the participants? | Were the outcomes or follow up results of cases clearly reported? | Was there clear reporting of the presenting site(s)/clinic(s) demographic information? | Was statistical analysis appropriate? |
| Draper | Low risk of bias | Low risk of bias | Low risk of bias | High risk of bias | High risk of bias | High risk of bias | High risk of bias | Low risk of bias | Low risk of bias | Low risk of bias |
| Jeong | Low risk of bias | Unclear risk of bias | Unclear risk of bias | Low risk of bias | Low risk of bias | Low risk of bias | High risk of bias | Low risk of bias | Low risk of bias | Low risk of bias |
| Han | Low risk of bias | Low risk of bias | Low risk of bias | Low risk of bias | Low risk of bias | Unclear risk of bias | High risk of bias | Low risk of bias | Low risk of bias | Low risk of bias |
| Burke | Low risk of bias | Low risk of bias | Low risk of bias | Low risk of bias | High risk of bias | Low risk of bias | High risk of bias | Low risk of bias | Low risk of bias | Low risk of bias |
| Ng | Low risk of bias | Low risk of bias | Low risk of bias | Low risk of bias | Low risk of bias | Low risk of bias | High risk of bias | Low risk of bias | Low risk of bias | Low risk of bias |
| Huang | Low risk of bias | Low risk of bias | Low risk of bias | Low risk of bias | Low risk of bias | High risk of bias | High risk of bias | Low risk of bias | Low risk of bias | Low risk of bias |
| Wang | Low risk of bias | Low risk of bias | Low risk of bias | Low risk of bias | Low risk of bias | Low risk of bias | High risk of bias | Low risk of bias | Low risk of bias | Low risk of bias |
| Jia | Low risk of bias | Low risk of bias | Low risk of bias | Low risk of bias | Low risk of bias | Low risk of bias | High risk of bias | Low risk of bias | Low risk of bias | Low risk of bias |
| Chen | Low risk of bias | Low risk of bias | Low risk of bias | Low risk of bias | Low risk of bias | Unclear risk of bias | High risk of bias | Low risk of bias | Low risk of bias | Low risk of bias |
| Luo | Low risk of bias | Low risk of bias | Low risk of bias | Low risk of bias | Low risk of bias | Low risk of bias | Low risk of bias | Low risk of bias | Low risk of bias | Low risk of bias |
| Bi | Low risk of bias | Low risk of bias | Low risk of bias | Unclear risk of bias | Unclear risk of bias | Low risk of bias | Low risk of bias | Low risk of bias | Low risk of bias | Low risk of bias |
| Liu | Low risk of bias | Low risk of bias | Low risk of bias | Low risk of bias | Low risk of bias | Unclear risk of bias | High risk of bias | Low risk of bias | Low risk of bias | Low risk of bias |
| Lei | Low risk of bias | Low risk of bias | Low risk of bias | Low risk of bias | Low risk of bias | Low risk of bias | High risk of bias | Low risk of bias | Low risk of bias | Low risk of bias |
| Li | Unclear risk of bias | Low risk of bias | Low risk of bias | Low risk of bias | Low risk of bias | High risk of bias | Low risk of bias | Low risk of bias | Low risk of bias | Low risk of bias |
| Bao | Low risk of bias | Low risk of bias | Low risk of bias | Low risk of bias | Low risk of bias | Low risk of bias | Low risk of bias | Low risk of bias | Low risk of bias | Low risk of bias |
| Xv | Low risk of bias | Low risk of bias | Low risk of bias | Low risk of bias | Low risk of bias | Low risk of bias | High risk of bias | Low risk of bias | Low risk of bias | Low risk of bias |
| Deng | Low risk of bias | Low risk of bias | Low risk of bias | Low risk of bias | Low risk of bias | High risk of bias | High risk of bias | Low risk of bias | Low risk of bias | Low risk of bias |
| Chen | Unclear risk of bias | Low risk of bias | Low risk of bias | Low risk of bias | Low risk of bias | Unclear risk of bias | Low risk of bias | Low risk of bias | Low risk of bias | Low risk of bias |
| Zhang | Low risk of bias | Low risk of bias | Low risk of bias | Low risk of bias | Low risk of bias | Low risk of bias | High risk of bias | Low risk of bias | Low risk of bias | Low risk of bias |
| Zhou | Low risk of bias | Low risk of bias | Low risk of bias | Low risk of bias | Low risk of bias | High risk of bias | Low risk of bias | Low risk of bias | Low risk of bias | Low risk of bias |
| Zhang | Low risk of bias | Low risk of bias | Low risk of bias | Low risk of bias | Low risk of bias | Low risk of bias | High risk of bias | Low risk of bias | Low risk of bias | Low risk of bias |
| Gao | Low risk of bias | Low risk of bias | Low risk of bias | Low risk of bias | Low risk of bias | Low risk of bias | Low risk of bias | Low risk of bias | Low risk of bias | Low risk of bias |
| Wu | Low risk of bias | Low risk of bias | Low risk of bias | Low risk of bias | Low risk of bias | Low risk of bias | High risk of bias | Low risk of bias | High risk of bias | Low risk of bias |
| Shen | Low risk of bias | Low risk of bias | Low risk of bias | Unclear risk of bias | Unclear risk of bias | Low risk of bias | Low risk of bias | Low risk of bias | Low risk of bias | Low risk of bias |
| Chen | Low risk of bias | Low risk of bias | Low risk of bias | Low risk of bias | Low risk of bias | Unclear risk of bias | High risk of bias | Low risk of bias | Low risk of bias | Low risk of bias |
| Wang | Low risk of bias | Low risk of bias | Low risk of bias | Low risk of bias | Low risk of bias | Unclear risk of bias | Low risk of bias | Low risk of bias | Low risk of bias | Low risk of bias |
| Zhao | Low risk of bias | Low risk of bias | Low risk of bias | Low risk of bias | Low risk of bias | Low risk of bias | High risk of bias | Low risk of bias | Low risk of bias | Low risk of bias |
| Akaishi | Low risk of bias | Low risk of bias | Low risk of bias | Low risk of bias | High risk of bias | Low risk of bias | High risk of bias | Low risk of bias | Low risk of bias | Low risk of bias |
| Al-Sakkaf | Low risk of bias | Low risk of bias | Low risk of bias | Low risk of bias | Low risk of bias | Low risk of bias | High risk of bias | Low risk of bias | Low risk of bias | Low risk of bias |
| Gettings | Low risk of bias | Low risk of bias | Low risk of bias | High risk of bias | Low risk of bias | Low risk of bias | Low risk of bias | Low risk of bias | Low risk of bias | Low risk of bias |
| Semakula | Low risk of bias | Low risk of bias | Low risk of bias | High risk of bias | High risk of bias | Low risk of bias | Low risk of bias | Low risk of bias | Low risk of bias | Low risk of bias |
| Sundar | Low risk of bias | Low risk of bias | Low risk of bias | High risk of bias | High risk of bias | Low risk of bias | High risk of bias | Low risk of bias | Low risk of bias | Low risk of bias |

**5. Supplementary table 5. Estimation of heterogeneity in pairwise comparisons**

|  |  | I2 | Q test | P |
| --- | --- | --- | --- | --- |
| All studies | A vs B | 93.50% | 201.35 | <0.001 |
|  | A vs C | 87.50% | 128.01 | <0.001 |
|  | A vs D | 88.70% | 132.91 | <0.001 |
|  | A vs E | 86.70% | 37.61 | <0.001 |
|  | A vs F | 86.60% | 104.38 | <0.001 |
|  | A vs G | 86.90% | 99.46 | <0.001 |
|  | B vs C | 70.70% | 17.09 | 0.004 |
|  | B vs D | 73.10% | 18.59 | 0.002 |
|  | B vs E | 84.90% | 26.5 | <0.001 |
|  | B vs F | 86.10% | 57.38 | <0.001 |
|  | B vs G | 10.10% | 5.56 | 0.352 |
|  | C vs D | 89.20% | 92.17 | <0.001 |
|  | C vs E | 45.60% | 5.52 | 0.138 |
|  | C vs F | 57.30% | 18.72 | 0.016 |
|  | C vs G | 4.00% | 7.29 | 0.399 |
|  | D vs E | 17.90% | 6.09 | 0.298 |
|  | D vs F | 10.60% | 10.07 | 0.345 |
|  | D vs G | 37.70% | 9.63 | 0.141 |
|  | E vs F | 65.90% | 14.65 | 0.012 |
|  | E vs G | 0.00% | 0.39 | 0.531 |
|  | F vs G | 0.00% | 5.01 | 0.756 |
| Studies from  mainland China | A vs B | 94.40% | 160.56 | <0.001 |
|  | A vs C | 88.30% | 120.04 | <0.001 |
|  | A vs D | 90.80% | 120 | <0.001 |
|  | A vs E | 80.20% | 15.16 | 0.002 |
|  | A vs F | 77.50% | 35.49 | <0.001 |
|  | A vs G | 82.00% | 49.99 | <0.001 |
|  | B vs C | 70.70% | 17.09 | 0.004 |
|  | B vs D | 69.00% | 9.69 | 0.021 |
|  | B vs E | 0.00% | 0.02 | 0.89 |
|  | B vs F | 16.20% | 5.97 | 0.309 |
|  | B vs G | 10.10% | 5.56 | 0.352 |
|  | C vs D | 90.10% | 90.81 | <0.001 |
|  | C vs E | 45.60% | 5.52 | 0.138 |
|  | C vs F | 40.70% | 11.8 | 0.107 |
|  | C vs G | 0.00% | 0.62 | 0.996 |
|  | D vs E | 0.00% | 1.51 | 0.681 |
|  | D vs F | 14.90% | 7.05 | 0.317 |
|  | D vs G | 47.50% | 9.53 | 0.09 |
|  | E vs F | 69.10% | 9.72 | 0.021 |
|  | E vs G | 0.00% | 0.39 | 0.531 |
|  | F vs G | 0.00% | 1.64 | 0.949 |
| Studies without small sample sizes | A vs B | 93.50% | 201.35 | <0.001 |
|  | A vs C | 88.70% | 124.09 | <0.001 |
|  | A vs D | 86.60% | 82.33 | <0.001 |
|  | A vs E | 86.70% | 37.61 | <0.001 |
|  | A vs F | 86.40% | 95.41 | <0.001 |
|  | A vs G | 87.30% | 94.22 | <0.001 |
|  | B vs C | 70.70% | 17.09 | 0.004 |
|  | B vs D | 73.10% | 18.59 | 0.002 |
|  | B vs E | 84.90% | 26.5 | <0.001 |
|  | B vs F | 86.10% | 57.38 | <0.001 |
|  | B vs G | 10.10% | 5.56 | 0.352 |
|  | C vs D | 82.00% | 38.85 | <0.001 |
|  | C vs E | 60.50% | 5.06 | 0.08 |
|  | C vs F | 57.30% | 18.72 | 0.016 |
|  | C vs G | 4.00% | 7.29 | 0.399 |
|  | D vs E | 32.70% | 5.94 | 0.203 |
|  | D vs F | 16.00% | 9.52 | 0.3 |
|  | D vs G | 47.50% | 9.53 | 0.09 |
|  | E vs F | 65.90% | 14.65 | 0.012 |
|  | E vs G | 0.00% | 0.39 | 0.531 |
|  | F vs G | 0.00% | 3.32 | 0.854 |
| Studies without low-quality | A vs B | 93.90% | 180.4 | <0.001 |
|  | A vs C | 87.50% | 128.01 | <0.001 |
|  | A vs D | 89.80% | 127.41 | <0.001 |
|  | A vs E | 89.50% | 38.06 | <0.001 |
|  | A vs F | 86.30% | 87.64 | <0.001 |
|  | A vs G | 86.90% | 99.46 | <0.001 |
|  | B vs C | 70.70% | 17.09 | 0.004 |
|  | B vs D | 69.00% | 9.69 | 0.021 |
|  | B vs E | 0.00% | 0.18 | 0.913 |
|  | B vs F | 16.20% | 5.97 | 0.309 |
|  | B vs G | 10.10% | 5.56 | 0.352 |
|  | C vs D | 89.20% | 92.17 | <0.001 |
|  | C vs E | 45.60% | 5.52 | 0.138 |
|  | C vs F | 57.30% | 18.72 | 0.016 |
|  | C vs G | 4.00% | 7.29 | 0.399 |
|  | D vs E | 0.00% | 1.51 | 0.681 |
|  | D vs F | 5.50% | 7.41 | 0.387 |
|  | D vs G | 37.70% | 9.63 | 0.141 |
|  | E vs F | 69.10% | 0.021 | 0.021 |
|  | E vs G | 0.00% | 0.39 | 0.531 |
|  | F vs G | 0.00% | 5.01 | 0.756 |

**6. Supplementary File 6. Estimation of inconsistency**

**6.1 Supplementary table 6. Estimation of inconsistency model**

|  | chi^2^ | *P* |
| --- | --- | --- |
| All 32 studies | chi^2^(55) = 89.57 | 0.0022 |
| 21 studies in mainland China | chi^2^(35) = 44.06 | 0.1401 |
| Sensitivity analysis for 27 studies (without small sample studies) | chi^2^(50) = 65.95 | 0.0759 |
| Sensitivity analysis for 28 studies (without low-quality studies) | chi^2^(46) = 77.01 | 0.0028 |

**6.2 The side-splitting (node-splitting) method and Loop-specific approach**

**A. All 32 studies**


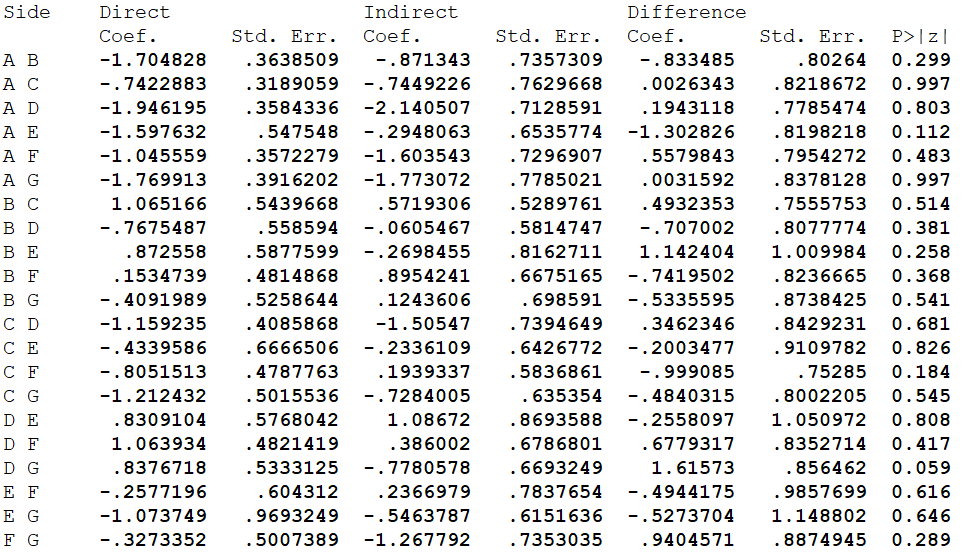

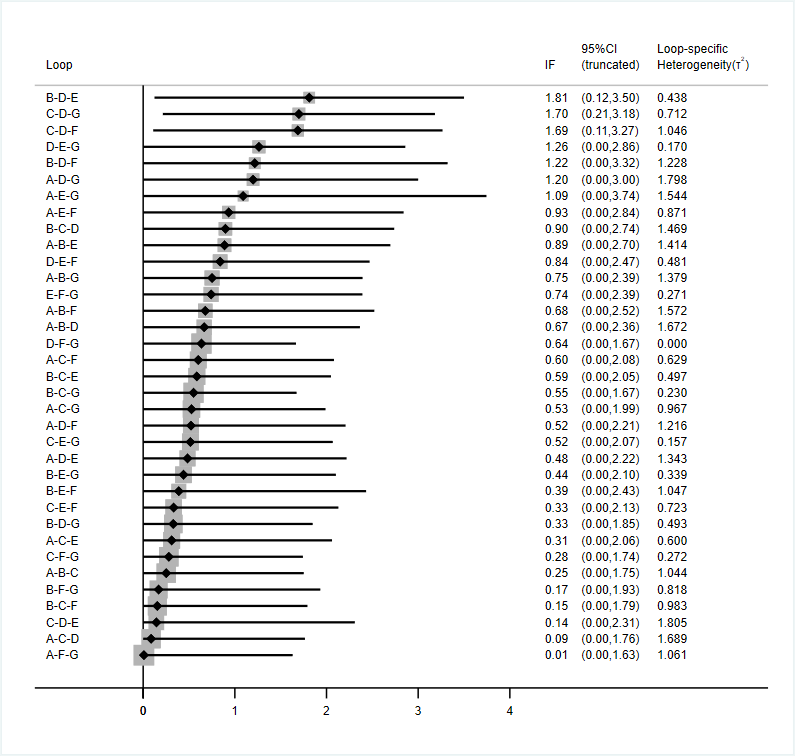


* All the evidence about these contrasts comes from the trials which directly compare them.

A=household settings. B=public places (entertainment, shopping, socializing, etc.). C=meal or gathering settings. D=transportation. E=daily conversation. F=work or study places. G=medical care.

**B. 21 studies in mainland China**


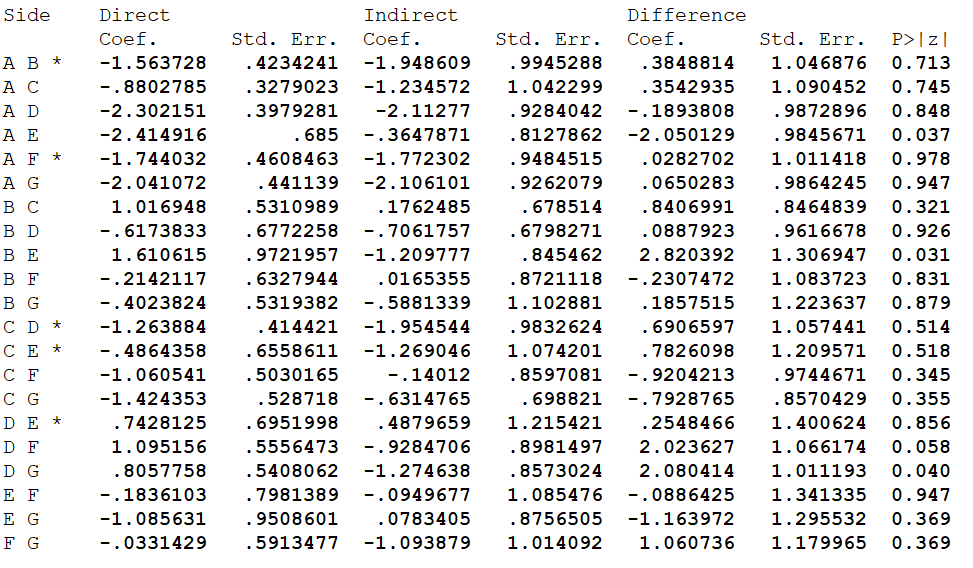

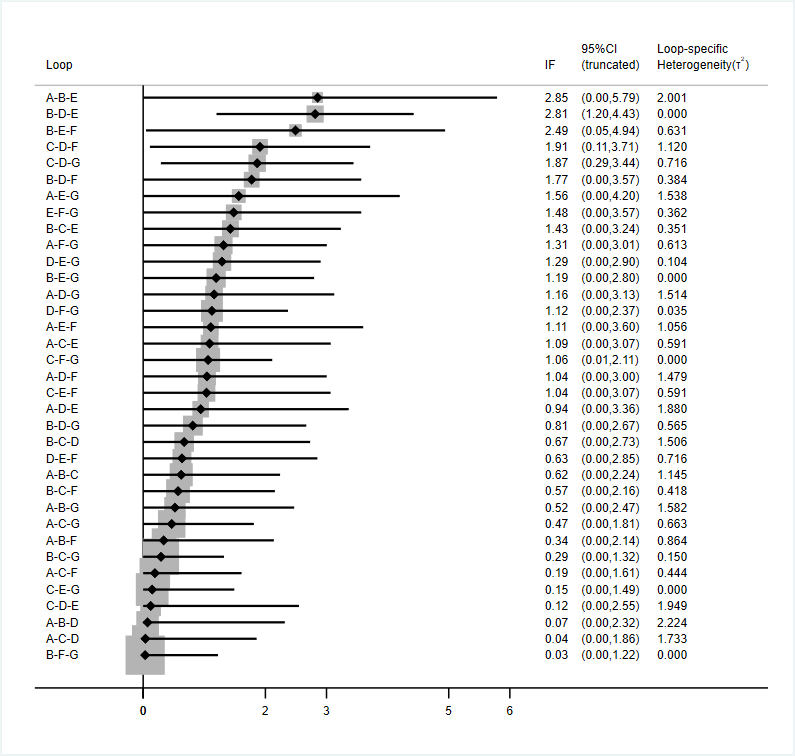


* All the evidence about these contrasts comes from the trials which directly compare them.

A=household settings. B=public places (entertainment, shopping, socializing, etc.). C=meal or gathering settings. D=transportation. E=daily conversation. F=work or study places. G=medical care.

**C. Sensitivity analysis for 27 studies (without small sample studies)**


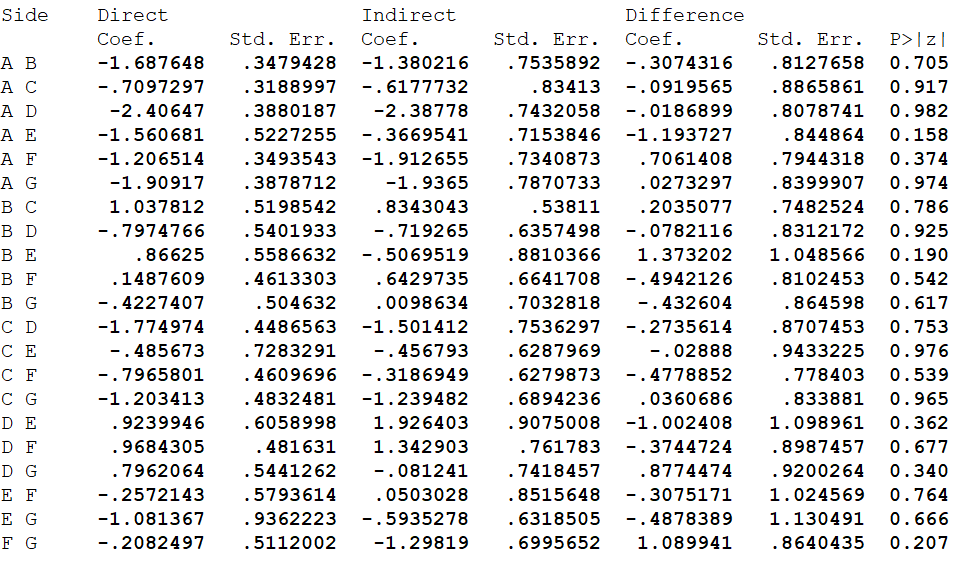

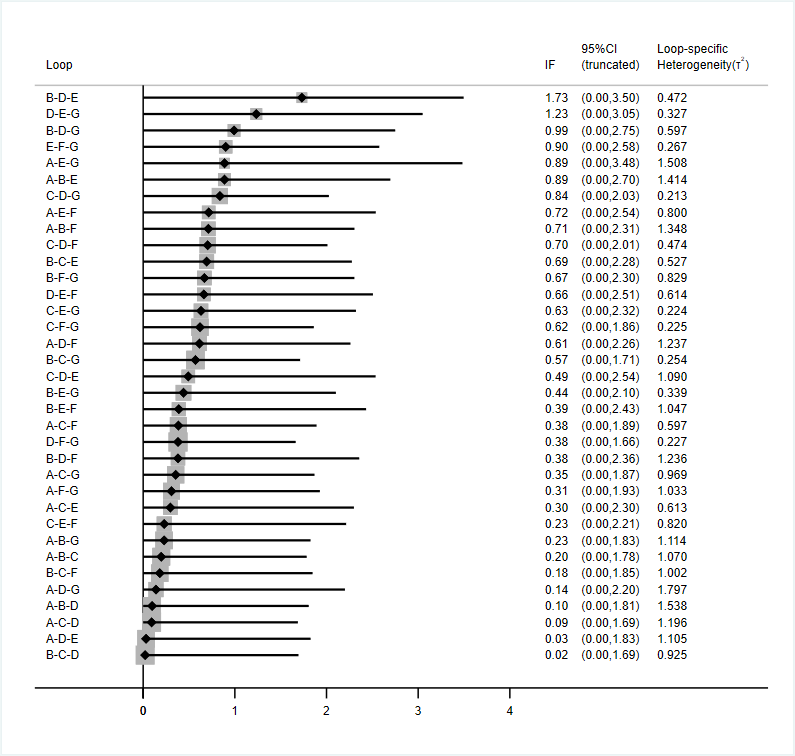


* All the evidence about these contrasts comes from the trials which directly compare them.

A=household settings. B=public places (entertainment, shopping, socializing, etc.). C=meal or gathering settings. D=transportation. E=daily conversation. F=work or study places. G=medical care.

**D. Sensitivity analysis for 26 studies (without poor-quality studies)**


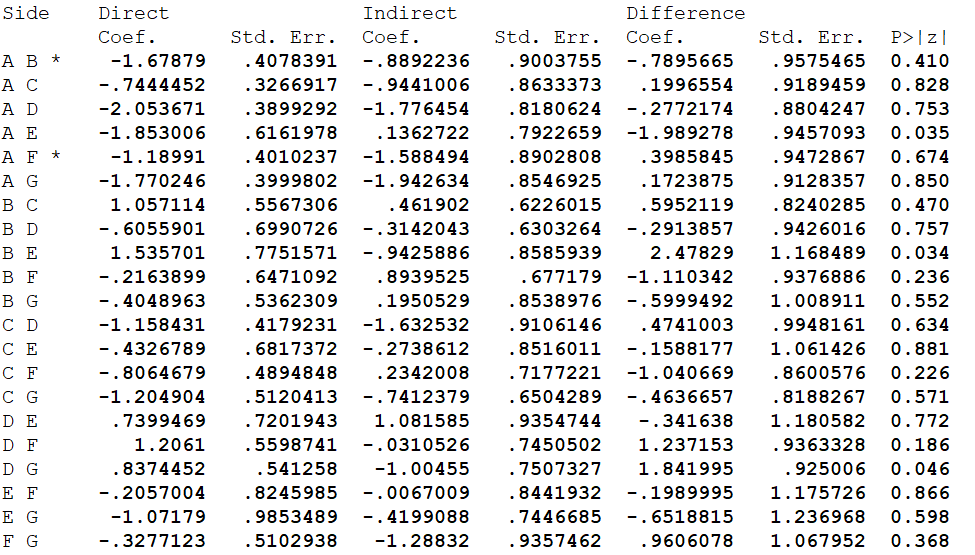

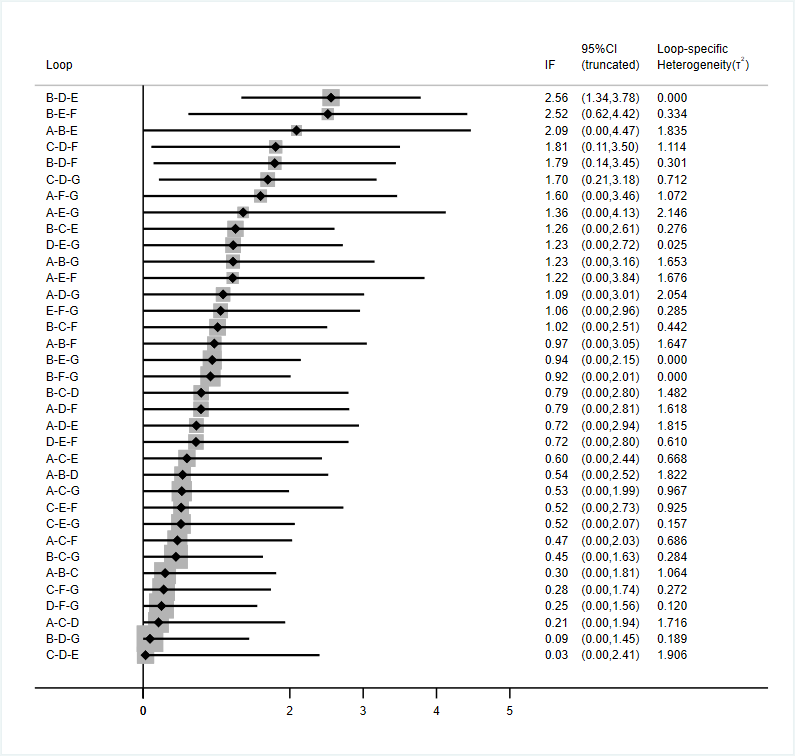


* All the evidence about these contrasts comes from the trials which directly compare them.

A=household settings. B=public places (entertainment, shopping, socializing, etc.). C=meal or gathering settings. D=transportation. E=daily conversation. F=work or study places. G=medical care.

**7. Supplementary File 7. SUCRA and cumulative probability plots**

**A. All 32 studies**


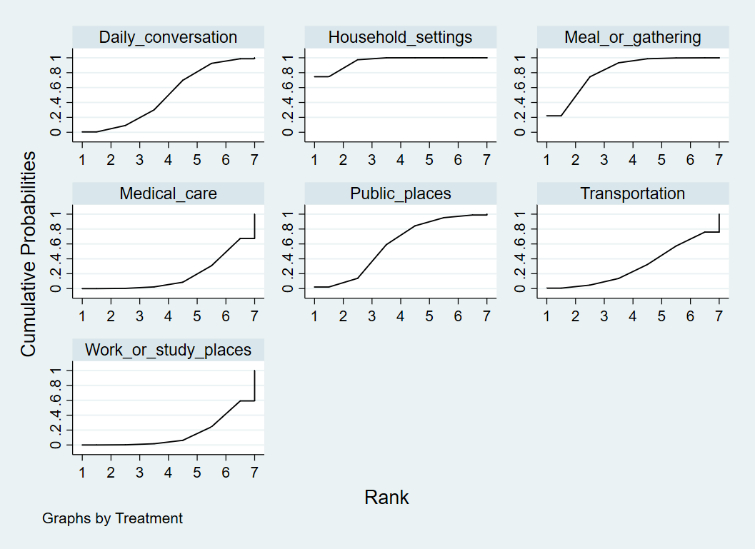

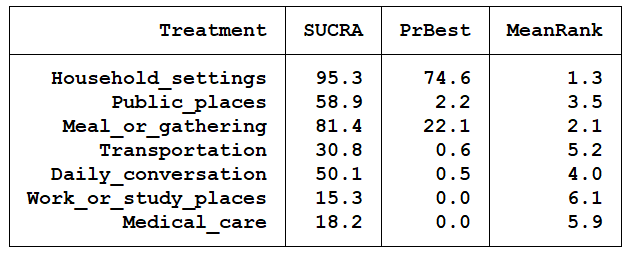


**B. 21 studies in mainland China**


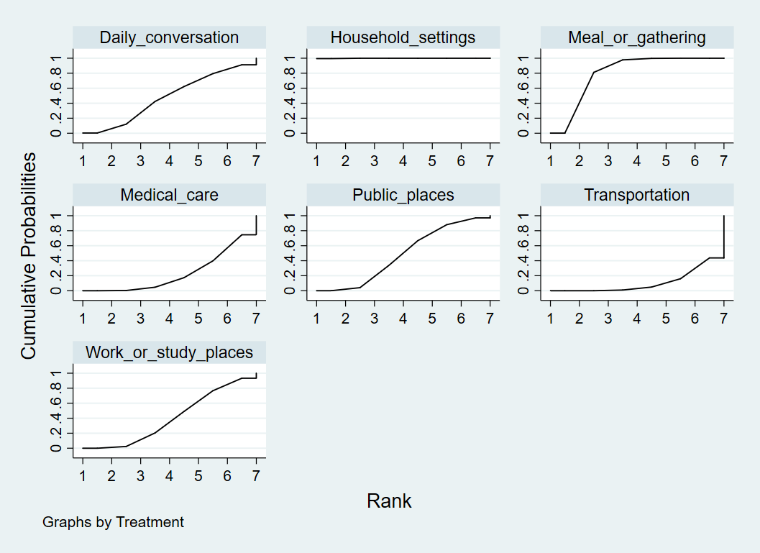

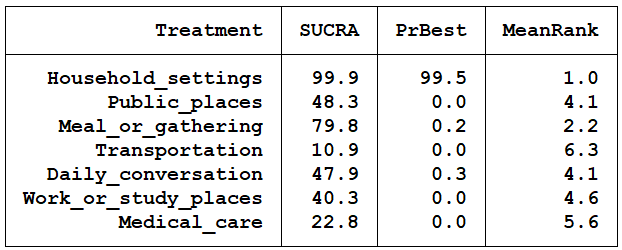


**C. Sensitivity analysis for 27 studies (without small sample studies)**


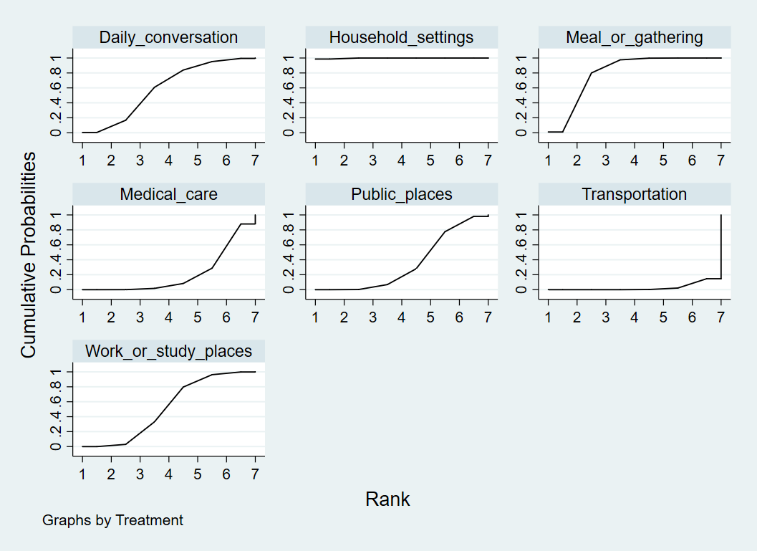

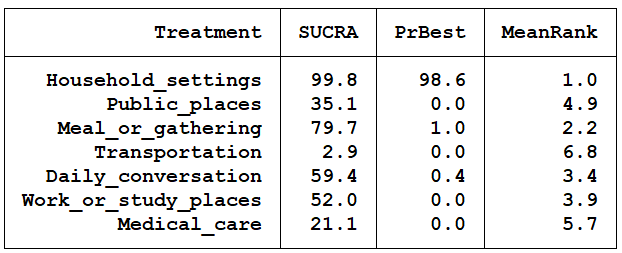


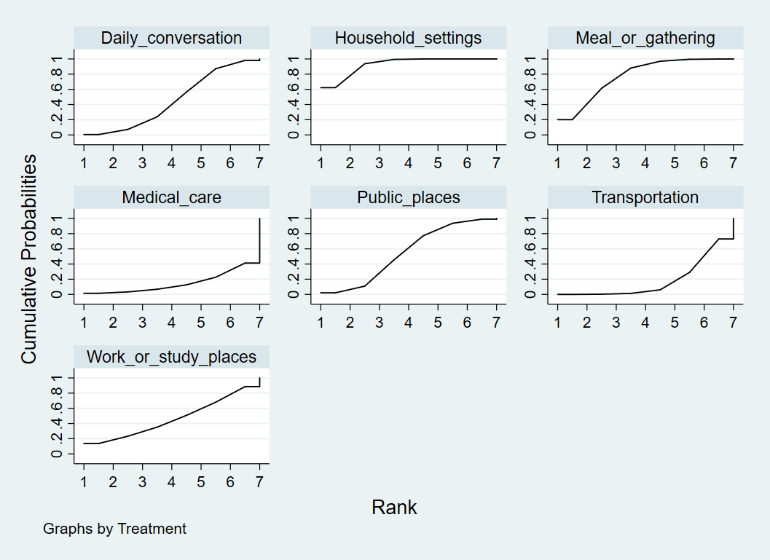

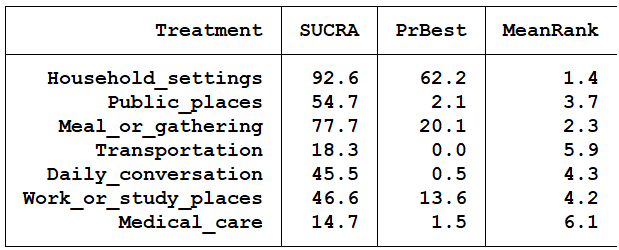
**D. Sensitivity analysis for 28 studies (without poor-quality studies)**

**8. Supplementary File 8. Forest plot of the effect estimates for pairwise comparison**

**A. All 32 studies**


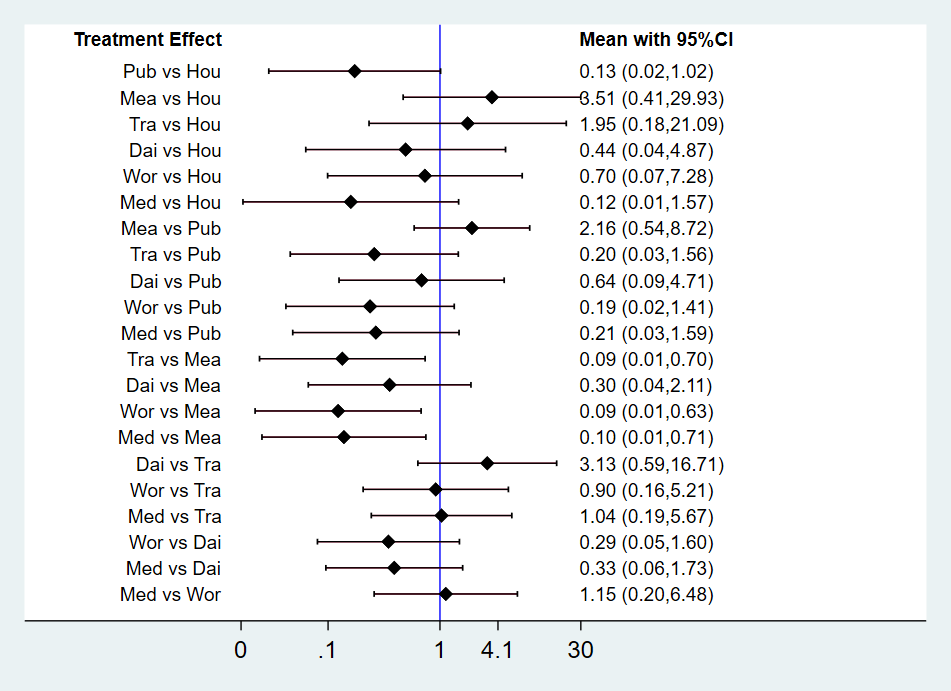


Hou=household settings. Pub=public places (entertainment, shopping, socializing, etc.). Mea=meal or gathering settings. Tra=transportation. Dai=daily conversation. Wor=work or study places. Med=medical care.

**B. 21 studies in mainland China**


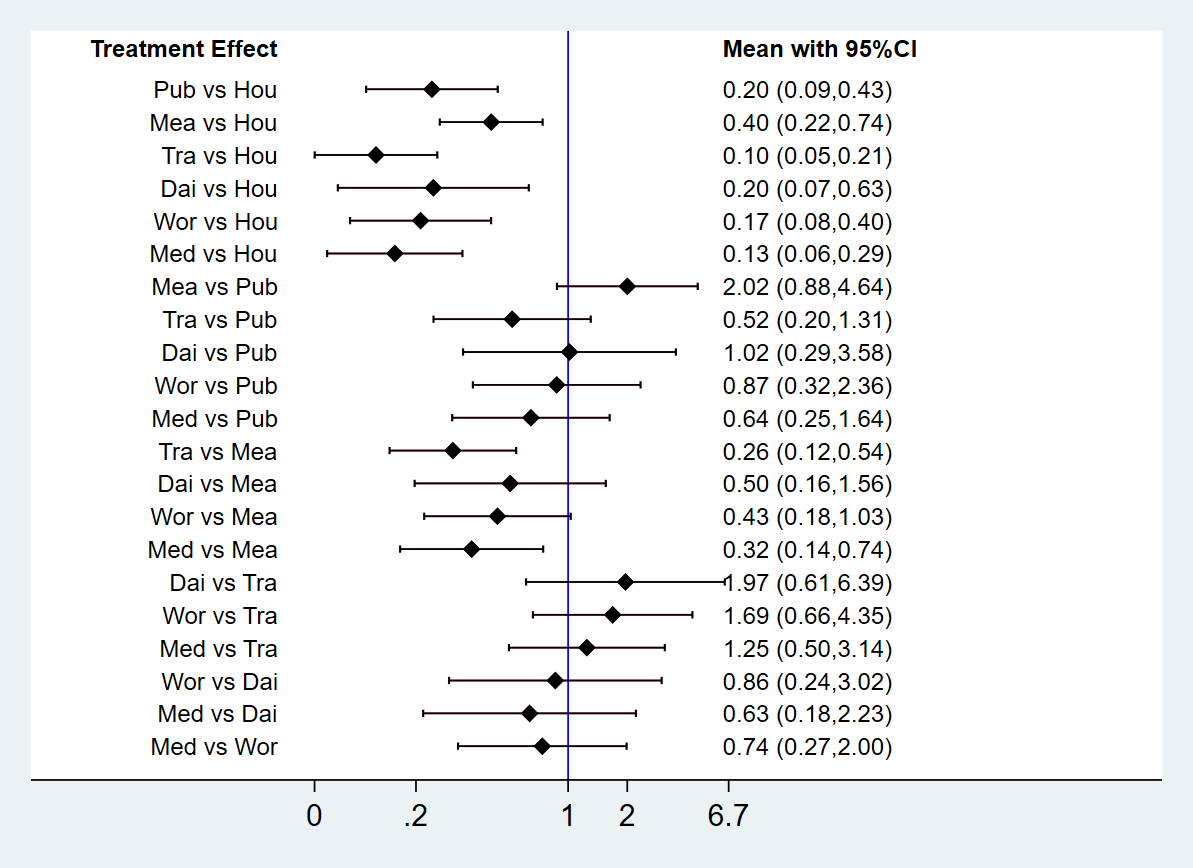


Hou=household settings. Pub=public places (entertainment, shopping, socializing, etc.). Mea=meal or gathering settings. Tra=transportation. Dai=daily conversation. Wor=work or study places. Med=medical care.

**C. Sensitivity analysis for 27 studies (without small sample studies)**


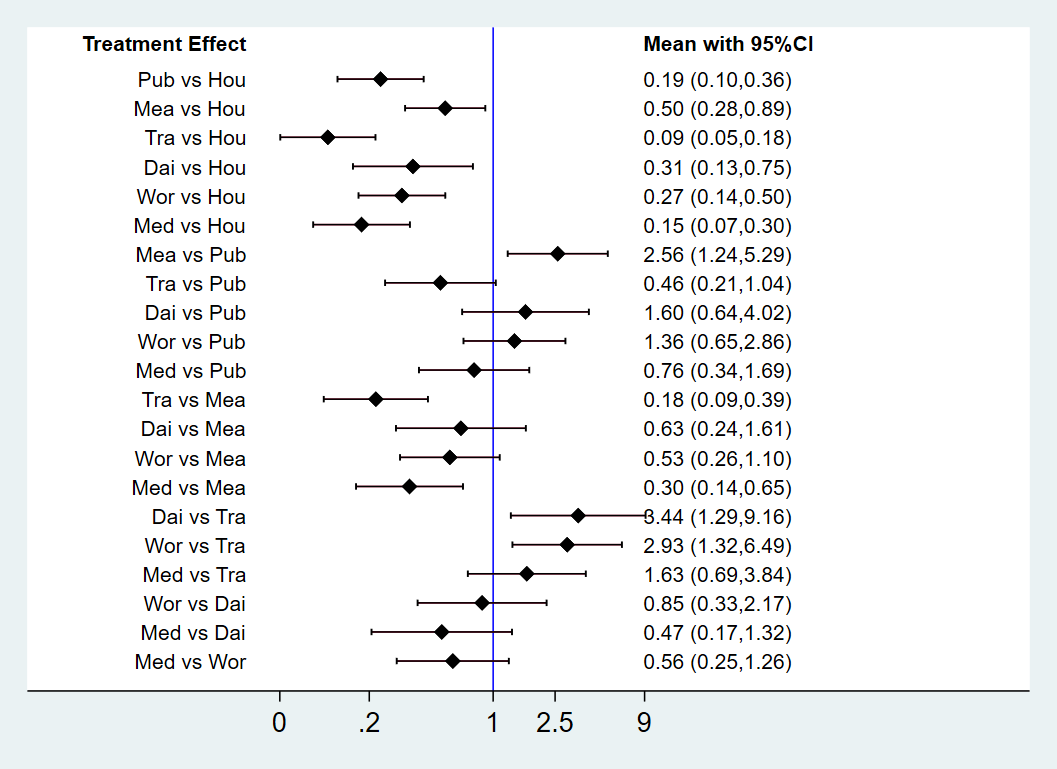


Hou=household settings. Pub=public places (entertainment, shopping, socializing, etc.). Mea=meal or gathering settings. Tra=transportation. Dai=daily conversation. Wor=work or study places. Med=medical care.

**D. Sensitivity analysis for 28 studies (without poor-quality studies)**


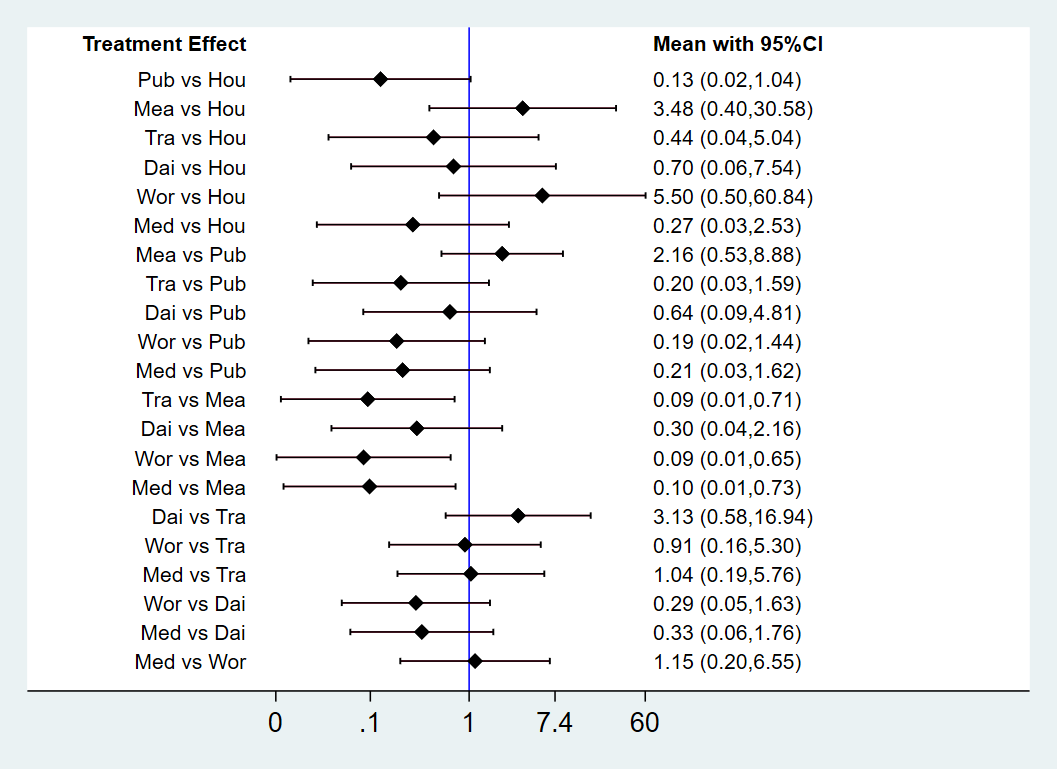


Hou=household settings. Pub=public places (entertainment, shopping, socializing, etc.). Mea=meal or gathering settings. Tra=transportation. Dai=daily conversation. Wor=work or study places. Med=medical care.

**9. Supplementary File 9. Funnel plots and Egger’s test**

**A. All 27 studies**

Egger’s test: *P*= 0.381


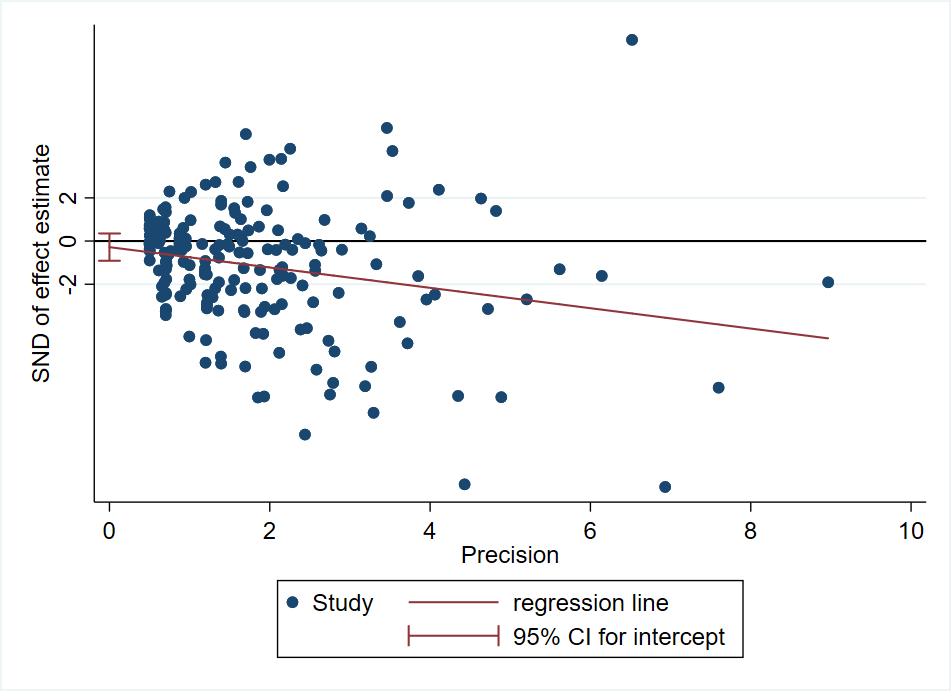

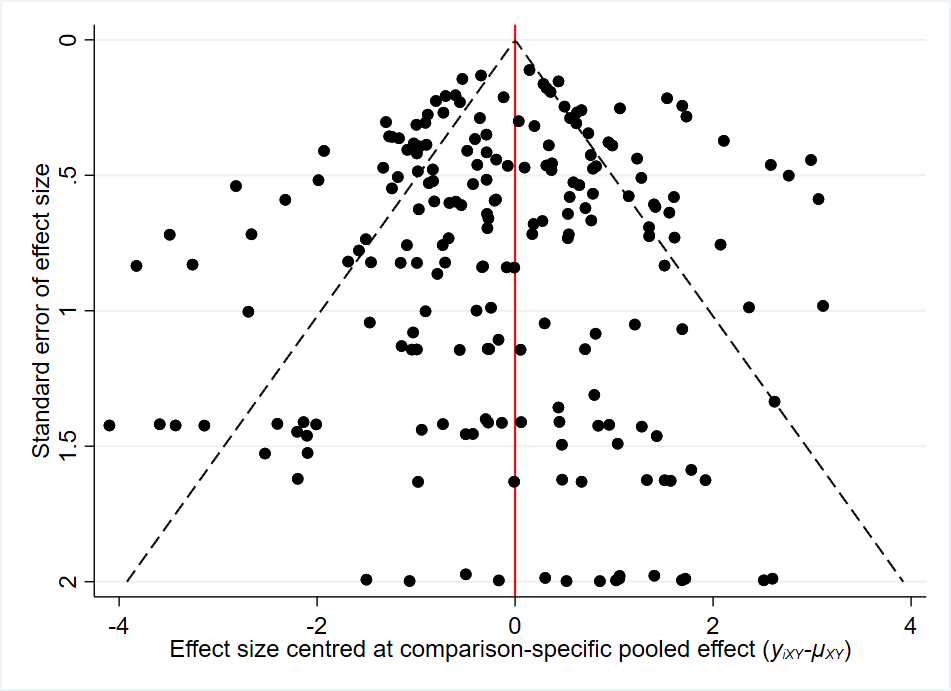


**B. 21 studies in mainland China**

Egger’s test: *P*= 0.601


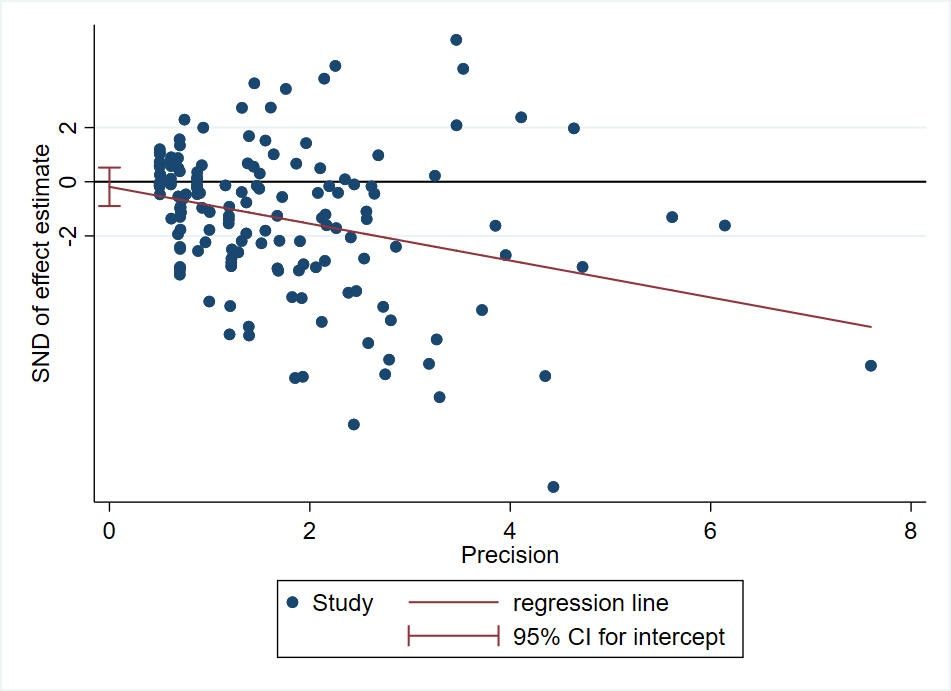

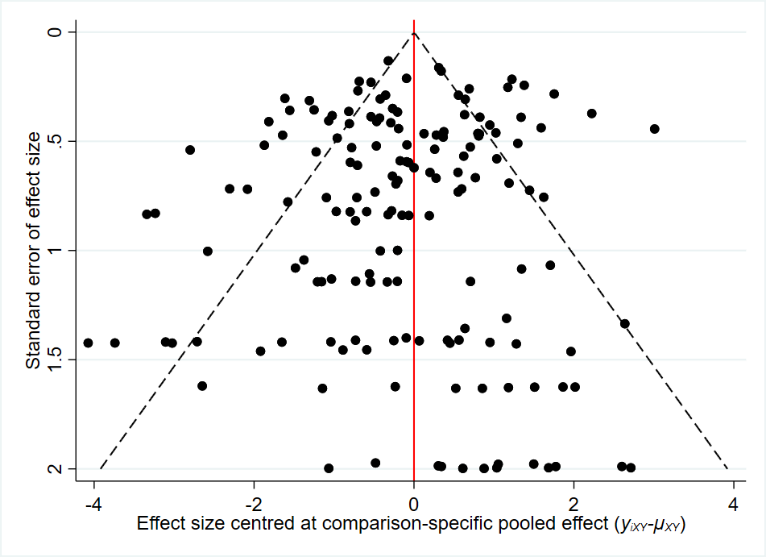


**C. Sensitivity analysis for 27 studies (without small sample studies)**

Egger’s test: *P*= 0.244


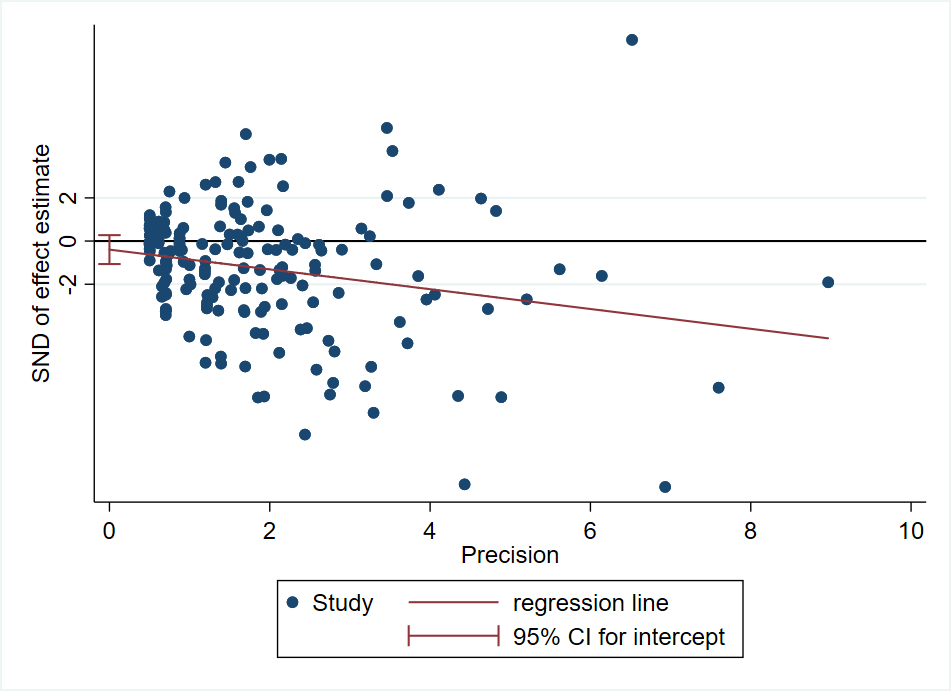

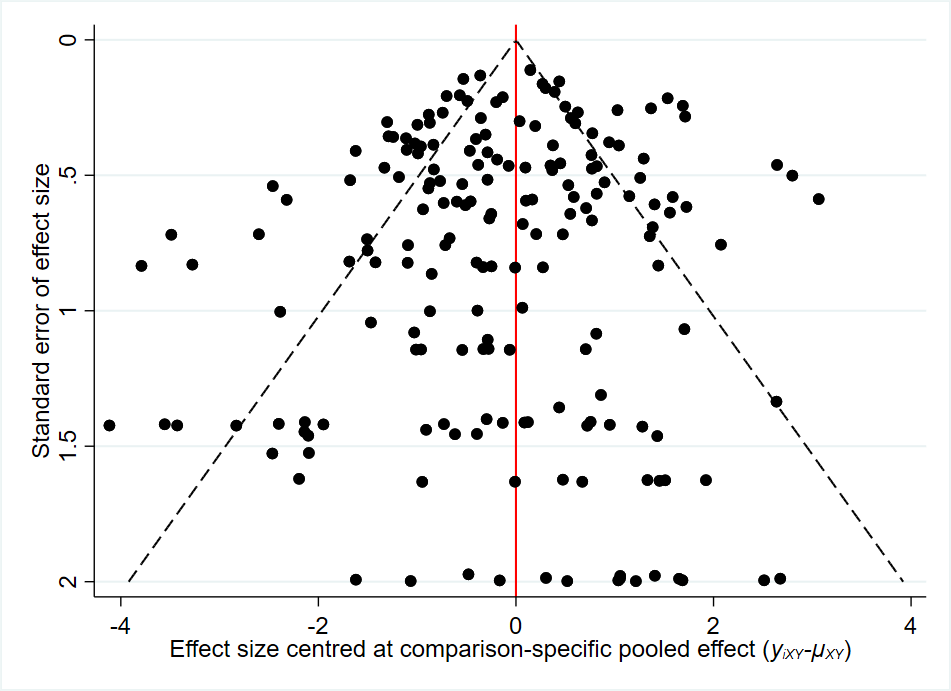


**D. Sensitivity analysis for 28 studies (without poor-quality studies)**

Egger’s test: *P*= 0.392


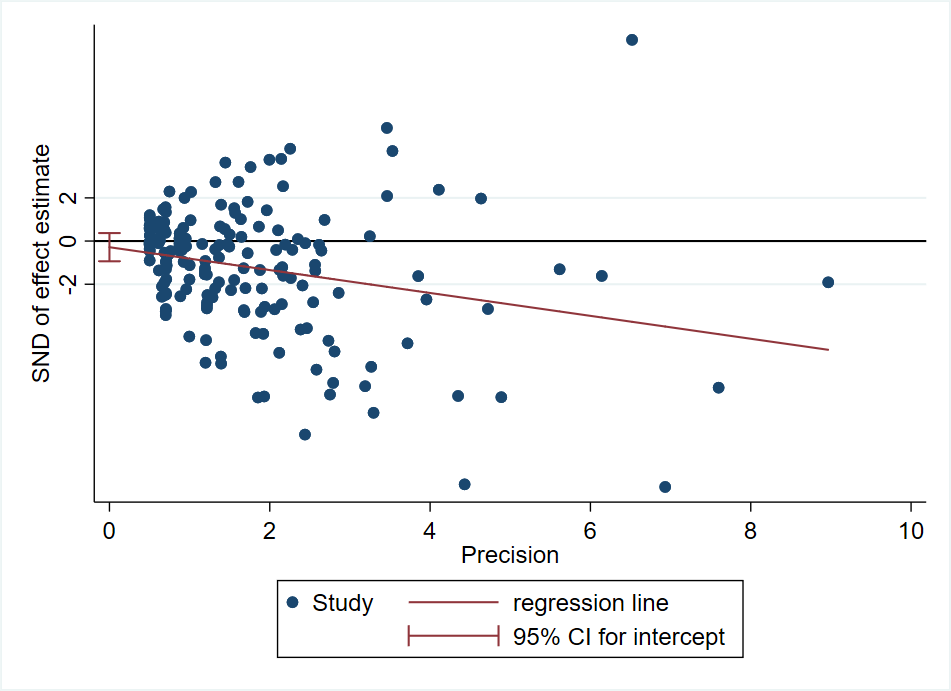

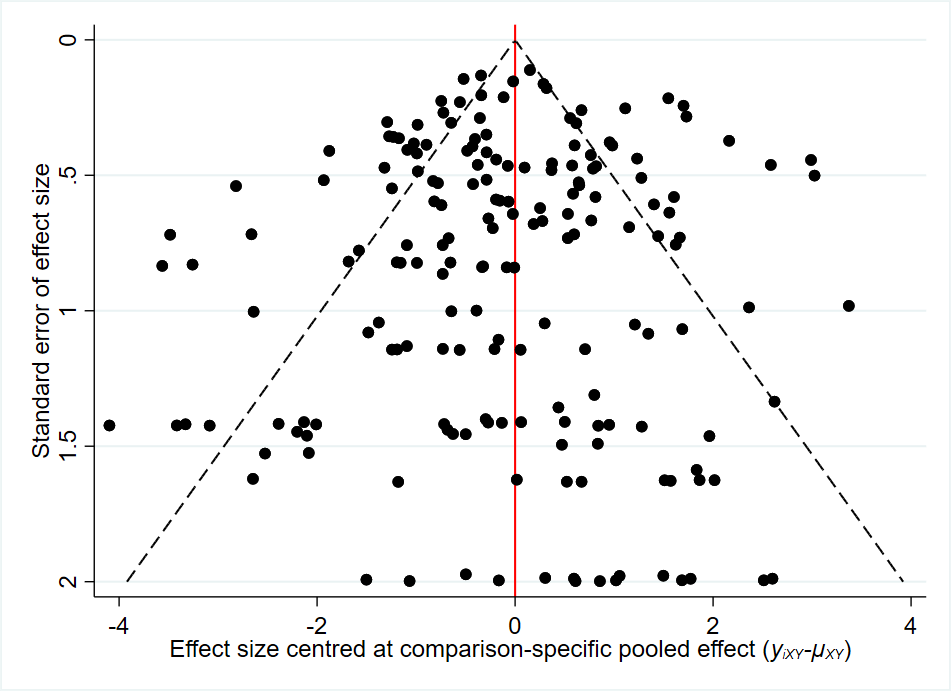

Supplement: Supplementary file 1 [file S0950268821002223sup001.docx]
